# Furanoditerpenes from *Spongia (Spongia) tubulifera* Display Mitochondrial-Mediated Neuroprotective Effects by Targeting Cyclophilin D

**DOI:** 10.1021/acschemneuro.2c00208

**Published:** 2022-07-28

**Authors:** Rebeca Alvariño, Amparo Alfonso, Dawrin Pech-Puch, Sandra Gegunde, Jaime Rodríguez, Mercedes R. Vieytes, Carlos Jiménez, Luis M. Botana

**Affiliations:** † Departamento de Farmacología, Facultad de Veterinaria, Universidad de Santiago de Compostela, 27002 Lugo, Spain; ‡ Grupo Investigación Biodiscovery, IDIS, 27002 Lugo, Spain; § Centro de Investigacións Científicas Avanzadas (CICA) e Departamento de Química, Facultade de Ciencias, Universidade da Coruña, 15071 A Coruña, Spain; ∥ Departamento de Biología Marina, Campus de Ciencias Biológicas y Agropecuarias, Facultad de Medicina Veterinaria y Zootecnia, Universidad Autónoma de Yucatán, 97100 Mérida, Yucatán, Mexico; ⊥ Fundación Instituto de Investigación Sanitario Santiago de Compostela (FIDIS), Hospital Universitario Lucus Augusti, 27002 Lugo, Spain; # Departamento de Fisiología, Facultad de Veterinaria, Universidad de Santiago de Compostela, 27002 Lugo, Spain

**Keywords:** furanoditerpenes, Spongia (Spongia) tubulifera, mitochondria, cyclophilin, neuroprotection, oxidative stress

## Abstract

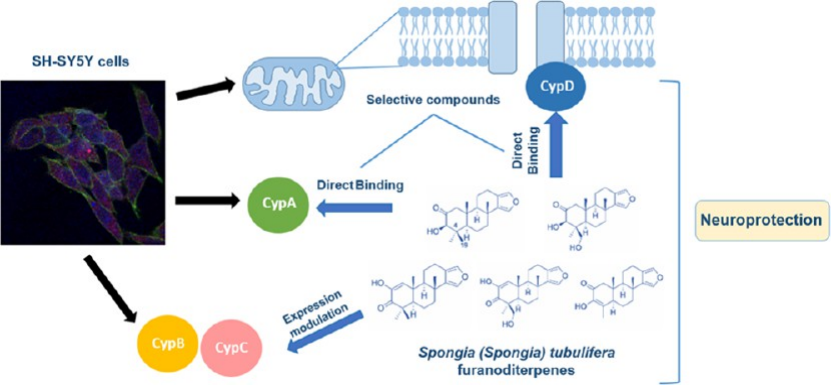

Neuroprotective properties of five previously described furanoditerpenes **1**–**5**, isolated from *Spongia (Spongia) tubulifera,* were evaluated in an *in vitro* oxidative stress model in SH-SY5Y cells. Dose–response treatments revealed that **1**–**5** improved cell survival at nanomolar concentrations through the restoration of mitochondrial membrane potential and the reduction of reactive oxygen species. Their ability to prevent the mitochondrial permeability transition pore opening was also assessed, finding that **4** and **5** inhibited the channel at 0.001 μM. This inhibition was accompanied by a decrease in the expression of cyclophilin D, the main regulator of the pore, which was also reduced by **1** and **2**. However, the activation of ERK and GSK3β, upstream modulators of the channel, was not affected by compounds. Therefore, their ability to bind cyclophilin D was evaluated by surface plasmon resonance, observing that **2**–**5** presented equilibrium dissociation constants in the micromolar range. All compounds also showed affinity for cyclophilin A, being **1** selective toward this isoform, while **2** and **5** exhibited selectivity for cyclophilin D. When the effects on the intracellular expression of cyclophilins A–C were determined, it was found that only **1** decreased cyclophilin A, while cyclophilins B and C were diminished by most compounds, displaying enhanced effects under oxidative stress conditions. Results indicate that furanoditerpenes **1**–**5** have mitochondrial-mediated neuroprotective properties through direct interaction with cyclophilin D. Due to the important role of this protein in oxidative stress and inflammation, compounds are promising drugs for new therapeutic strategies against neurodegeneration.

## 1 Introduction

Sponges are the most studied organisms for the discovery of active natural products, many of them showing anti-inflammatory, immunosuppressant, or antibiotic properties.^1^ Specifically, the genus *Spongia* has been a prolific source of different compounds such as terpenes, macrolides, and sterols,^2^ being furanoterpenes, spongian diterpenes, and scalarane sesterterpenoids the most common structures obtained from this genus.^1^

Prevention of mitochondrial dysfunction has been proposed as a therapeutic strategy against several diseases. Age-related illnesses are closely associated with this organelle dysfunction and, therefore, with an increase in the release of damaging reactive oxygen species (ROS). Since mitochondria are responsible for oxidative phosphorylation, they are the main ROS producers when the electronic transport chain begins to fail.^3,4^ This generation directly affects the organelle, which is the principal target of these damaging species, inducing a dangerous feedback. ROS accumulation produces the opening of the mitochondrial permeability transition pore (mPTP). The pore is a large nonspecific channel that connects the mitochondrial matrix and the cytosol and allows the passage of solutes with a molecular weight of less than 1500 Da, provoking an increase in the permeability of the inner mitochondrial membrane. mPTP opening is reversible, providing a pathway to release ROS and calcium from mitochondria, but if it is massive and persistent, it can lead to mitochondrial swelling and cell death.^5,6^

The molecular nature of the pore is still an open question. For a long time, mPTP was considered to be composed of adenine nucleotide translocase (ANT) in the inner membrane and voltage-dependent anion channel (VDAC) in the outer membrane, but their genetic ablation did not prevent pore opening.^7^ Although ANT is still considered a part of the pore, F-ATP synthase has attracted attention as the main component of mPTP.^6^ In any case, the best-accepted regulator of the pore is cyclophilin D (CypD). This protein is the only mitochondrial isoform of a family with more than 15 members that present the peptidyl–prolyl isomerase activity and share the capacity to bind to cyclosporine A (CsA). The best-known isoforms include cyclophilin A (CypA), involved in the CsA immunosuppressant activity, and cyclophilins B (CypB) and C (CypC), implicated in endoplasmic reticulum homeostasis.^8^ These enzymes have been associated with aging, inflammatory processes, and ROS augmentation.^9^ In fact, the role of CypD in mitochondria goes beyond mPTP modulation; it has been described as a regulator of the organelle proteome and function.^10^

Due to the high dependence on the energy consumption of neurons, these cells are very sensitive to mitochondrial malfunction and oxidative damage. In this context, neurodegenerative diseases have been associated with these pathological mechanisms, and therefore, mPTP opening and CypD have been proposed as important targets for counteracting these devastating illnesses.^7,11^

**Figure 1:**
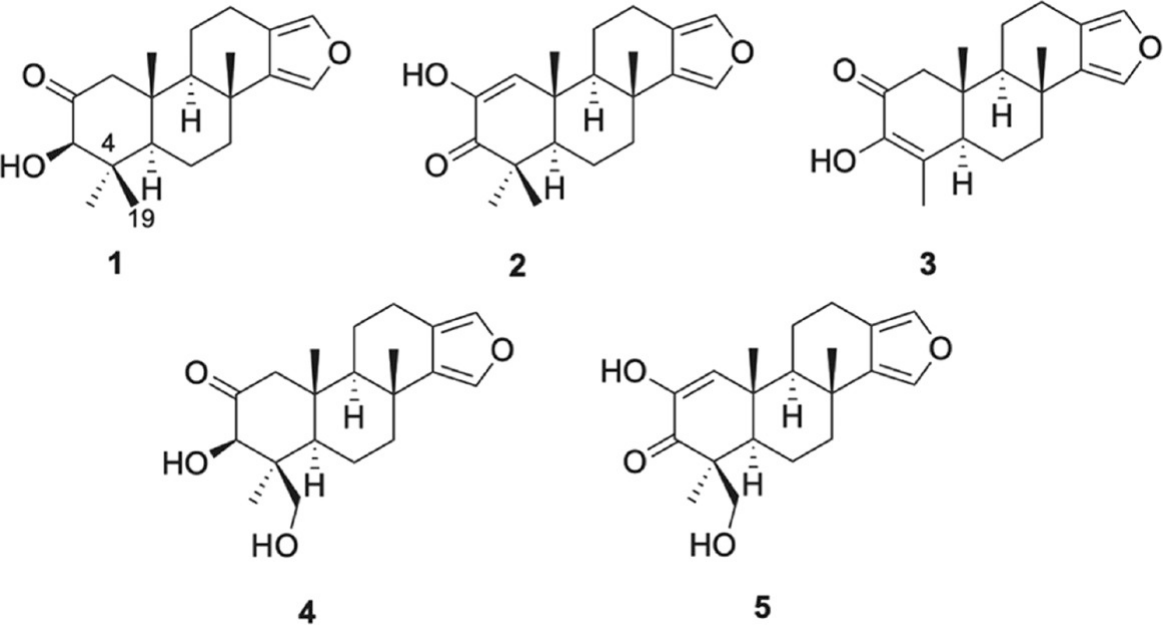
Chemical structures of furanoditerpenes **1**–**5**.

In this work, five furanoditerpenes (**1**–**5**) (Figures 1 and S1–S5) isolated from the sponge *Spongia (Spongia) tubulifera*,^2^ including epispongiadiol (**4**)^12,13^ and spongian diterpene 17 (**5**),^14^ were tested in SH-SY5Y human neuroblastoma cells to determine their neuroprotective activity. In previous studies, compounds **1** and **4** displayed weak cytotoxicity in human cancer cell lines,^2^ and **4** presented antiviral activity,^12^ but their neuroprotective effects have not been tested so far.

## 2 Results and Discussion

### 2.1 Compounds Protect Neuronal Cells from Oxidative Injury

Before the beginning of neuroprotection assays, cytotoxic effects of diterpenes **1**–**5** on SH-SY5Y cells were evaluated. Metabolites were added at concentrations ranging from 0.001 to 1 μM to neuroblastoma cells for 24 h, and cell viability was analyzed with the 3-(4,5-dimethylthiazol-2-yl)-2,5-diphenyltetrazolium bromide (MTT) test. Compounds **1**–**5** did not display toxicity at any of the doses tested (data not shown). Therefore, neuroprotective experiments were performed with *S. tubulifera* metabolites at 0.001, 0.01, 0.1, and 1 μM. Oxidative damage was induced by adding 150 μM H_2_O_2_ for 6 h since this concentration has been previously shown to significantly reduce cell viability, affect mitochondrial function, and increase the ROS release, mimicking the pathological effects observed in neurodegenerative illnesses.^15^

**Figure 2:**
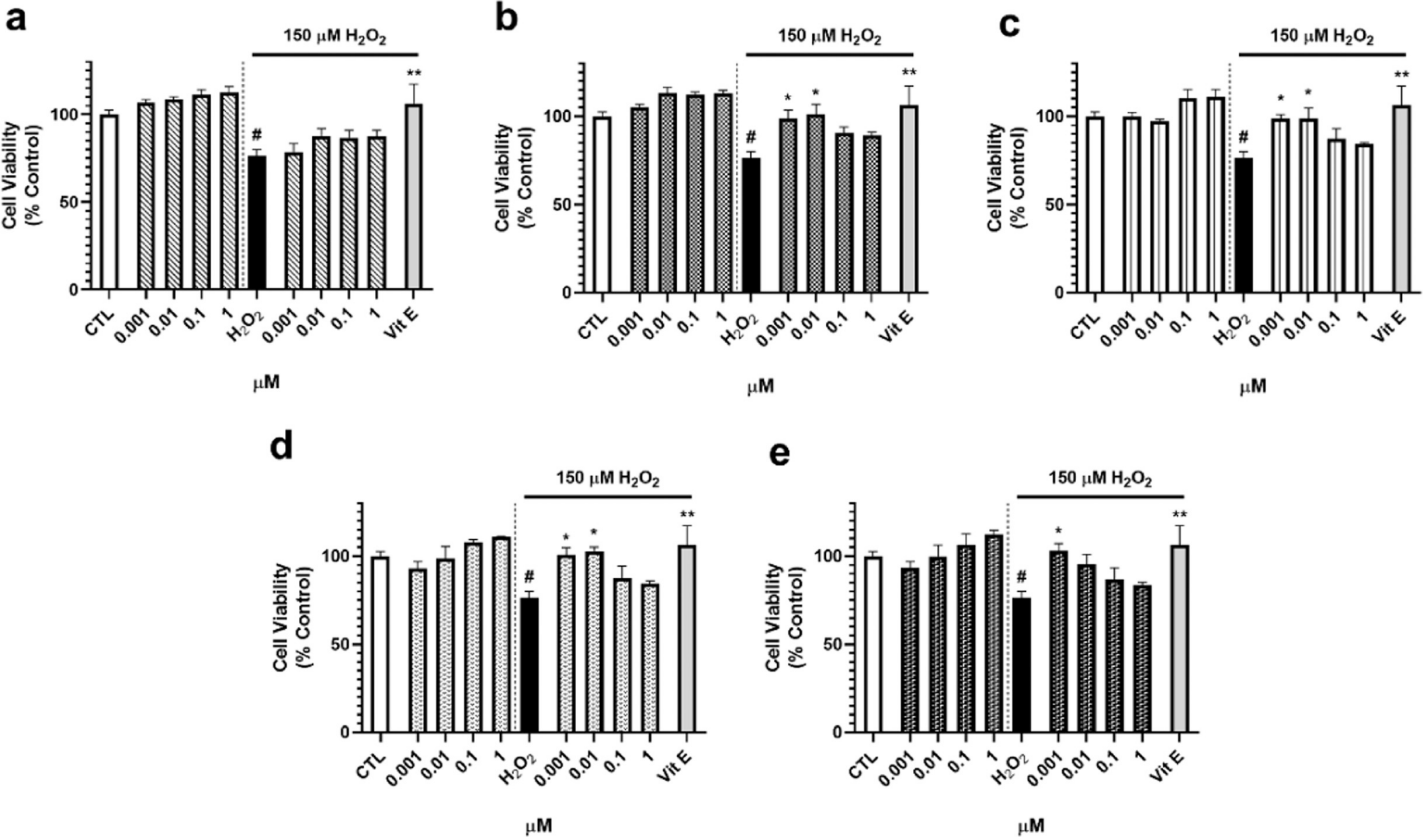
Effect of compounds on the cell viability of SH-SY5Y cells. Compounds were added to human neuroblastoma cells for 6 h with and without 150 μM H_2_O_2_, and cell viability was evaluated with the MTT assay. Results of (a) compound **1**, (b) compound **2**, (c) compound **3**, (d) compound **4**, and (e) compound **5**. Vitamin E (Vit E) at 25 μM was used as a positive control in oxidative stress assays. Mean ± standard error of the mean (SEM) of three experiments performed in triplicate. Data expressed as the percentage of control cells, compared by one-way analysis of variance (ANOVA) and Dunnett’s tests. ^#^*p* < 0.05 compared to untreated control cells. *p < 0.05 and **p < 0.01 compared to cells treated with H_2_O_2_ alone.

First, the capacity of **1**–**5** to improve cell survival under oxidative stress conditions was determined with the MTT assay (Figure 2). As expected, treatment with compounds alone for 6 h did not affect cell viability. When cells were injured with the oxidant specie, a decrease in the cell viability of 23.6 ± 3.5% (*p* < 0.05) was observed. Compound **1** was not able to protect neuroblastoma cells from this damage (Figure 2a). However, diterpenes **2**–**5** significantly ameliorated cell survival at the lowest concentrations used, reaching levels among 95.5–101.2% (*p* < 0.05), similar to the effect observed with the positive control at 25 μM (Figure 2b–e).

**Figure 3:**
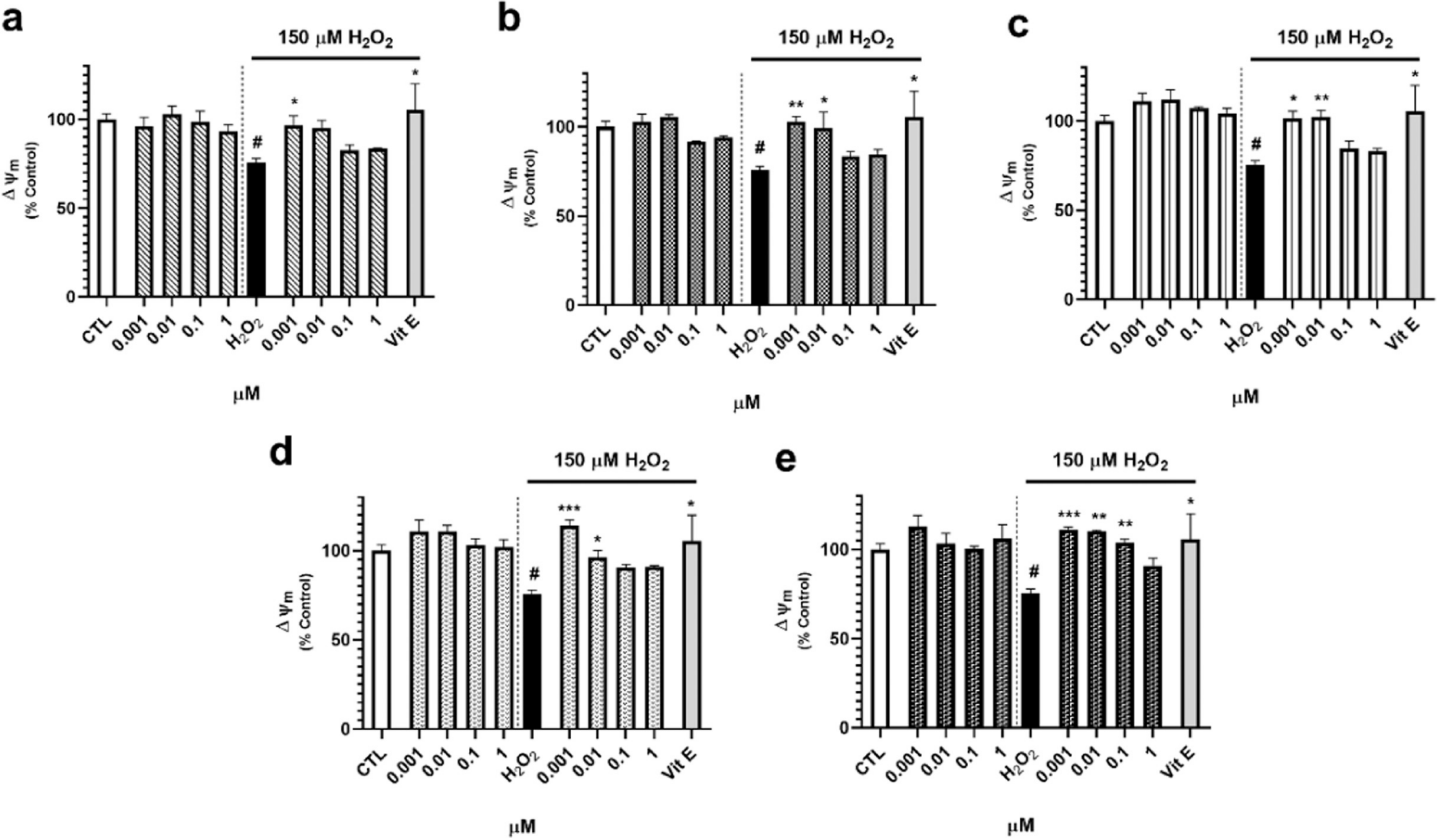
Effect of furanoditerpenes on ΔΨ_m_ of human neuroblastoma cells. Cells were treated for 6 h with *S. tubulifera* metabolites with and without 150 μM H_2_O_2_, and ΔΨ_m_ was determined with the TMRM dye. (a) Compound **1**, (b) compound **2**, (c) compound **3**, (d) compound **4**, and (e) compound **5** effects. Vitamin E (Vit E) at 25 μM was used as a positive control in oxidative stress assays. Data expressed as the percentage of control cells. Mean ± SEM of three experiments performed in triplicate. Statistical differences determined by one-way ANOVA and Dunnett’s tests. ^#^*p* < 0.05 compared to untreated control cells. *p < 0.05, **p < 0.01, and ***p < 0.01 compared to cells treated with H_2_O_2_ alone.

Since mitochondria are key players in neurodegeneration, acting as main producers and targets of ROS,^16^ mitochondrial function was determined by analyzing the mitochondrial membrane potential (ΔΨ_m_) with the fluorescent dye tetramethylrhodamine methyl ester (TMRM). As Figure 3 shows, furanoditerpenes **1**–**5** did not produce effects on ΔΨ_m_ when added alone to SH-SY5Y cells, but all of them were capable of recovering mitochondria from the depolarization induced by H_2_O_2_ (75.6 ± 2.3%, *p* < 0.05). Compound **1** improved ΔΨ_m_ after treatment at 0.001 μM (96.7 ± 5.4%, *p* < 0.05) (Figure 3a), whereas **2**–**4** repolarized the organelle at 0.001 and 0.01 μM (Figure 3b–d). Compound **5** was the most effective one, increasing ΔΨ_m_ at 0.001, 0.01, and 0.1 μM, with levels among 111.3 and 98.0% of control cells (Figure 3e).

**Figure 4:**
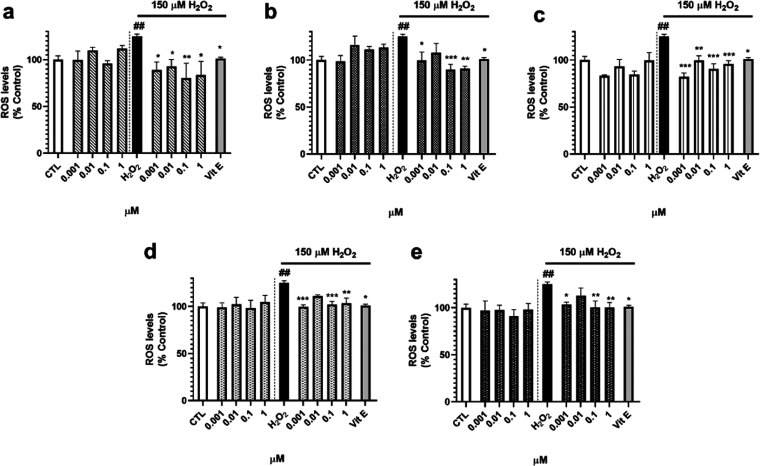
ROS intracellular levels after treatment with compounds. Furanoditerpenes were added for 6 h in the absence and presence of 150 μM H_2_O_2_. Then, the ROS content was measured with the fluorescent dye 5-(and-6)-carboxy-2′,7′-dichlorodihydrofluorescein diacetate (carboxy-H_2_DCFDA). Results from (a) **1**, (b) **2**, (c) **3**, (d) **4**, and (e) **5**. Vitamin E (Vit E) at 25 μM was used as a positive control. Data expressed as the percentage of untreated control cells. Mean ± SEM of three experiments performed in triplicate. Statistical differences determined by one-way ANOVA test followed by Dunnett’s post hoc test. ^##^*p* < 0.01 compared to control cells. *p < 0.05, **p < 0.01, and ***p < 0.01 compared to H_2_O_2_ control.

Next, the capacity of *S. tubulifera* metabolites to decrease ROS intracellular levels was evaluated (Figure 4). Once again, treatment with compounds alone did not modify ROS levels in human neuronal cells. Regarding the oxidative stress model, H_2_O_2_ augmented the levels of oxidant molecules up to 125.1 ± 2.4% (*p* < 0.01) and the known antioxidant Vit E, used as a positive control, induced a reduction in ROS release of 24% with respect to cells treated only with H_2_O_2_ (*p* < 0.05). Cotreatment with **1** produced a significant decrease in the ROS content at all of the doses assayed (Figure 4a). Compound **2** at 0.001, 0.1, and 1 μM also reduced the levels of these damaging species, reaching a percentage of 89.9 ± 5.7% at 0.1 μM (*p* < 0.001) (Figure 4b). With respect to **3**, this compound diminished ROS levels at the four concentrations tested (Figure 4c). Finally, diterpenes **4** and **5** were also capable of decreasing the intracellular content of ROS at 0.001, 0.1, and 1 μM, with levels between 99.6 and 103.4% of untreated control cells (Figure 4d,e).

**Figure 5:**
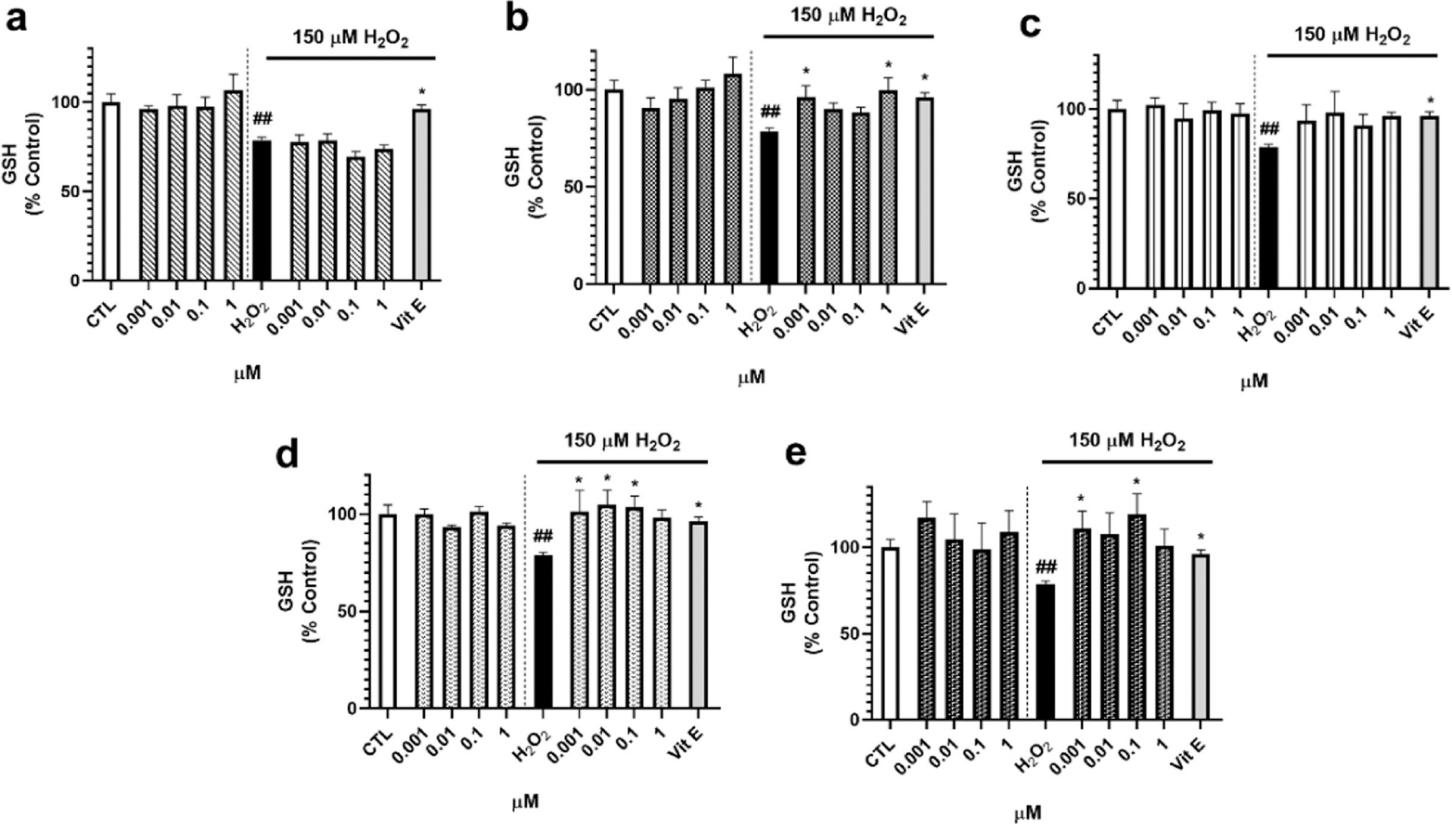
Effect of *S. tubulifera* metabolites in the GSH content of human neuroblastoma cells. SH-SY5Y cells were treated with compounds for 6 h with and without 150 μM H_2_O_2_. GSH levels were monitored with Thiol Tracker Violet dye. (a) Compound **1**, (b) compound **2**, (c) compound **3**, (d) compound **4**, and (e) compound **5** results. Vitamin E (Vit E) at 25 μM was used as a positive control. Mean ± SEM of three experiments performed in triplicate. Data presented as the percentage of untreated control cells and compared by one-way ANOVA and Dunnett’s tests. ^##^*p* < 0.01 compared to control cells. *p < 0.05 compared to cells treated with H_2_O_2_ alone.

To confirm the antioxidant properties of *S. tubulifera* compounds, the levels of the most important nonenzymatic antioxidant of cells, glutathione (GSH), were assessed. Treatment with **1** did not affected the GSH content neither with the compound alone nor under oxidative damage (Figure 5a). Regarding diterpene **2**, an increase in the nonenzymatic antioxidant was observed when cells were cotreated with H_2_O_2_ and this compound at 0.001 and 1 μM (*p* < 0.05) (Figure 5b). No effects were found after the addition of **3** (Figure 5c), but **4** and **5** presented interesting results (Figure 5d,e). Compound **4** increased the GSH content at the lowest concentrations tested until control cell levels (101.3–105.0%), and **5** recovered the endogenous antioxidant at 0.001 (110.9 ± 10.0%, *p* < 0.05) and 0.1 μM (118.9 ± 12.0%, *p* < 0.05) compared to H_2_O_2_ control cells.

### 2.2 Effect of Furanoditerpenes **1**–**5** on Mitochondrial Permeability Transition Pore

**Figure 6:**
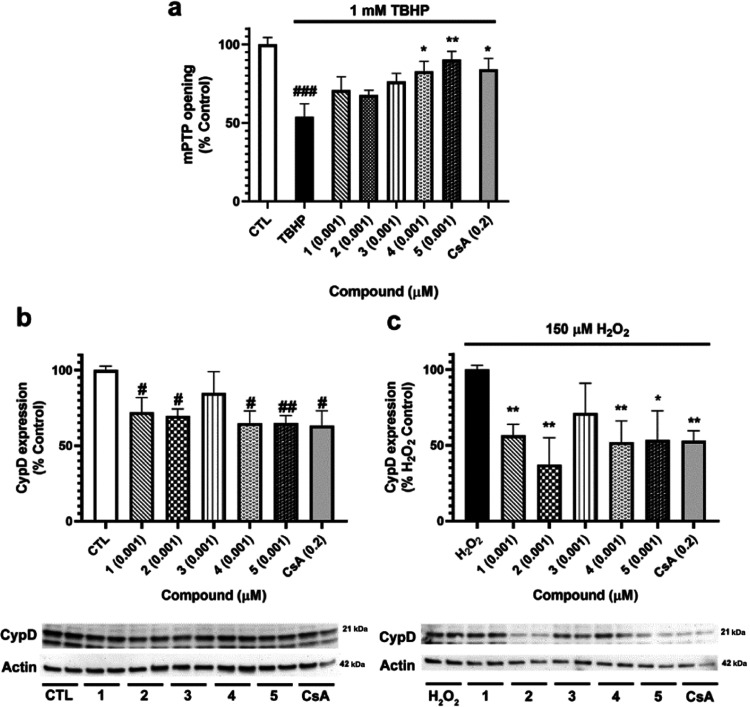
Evaluation of mPTP in SH-SY5Y cells treated with furanoditerpenes. (a) Analysis of mPTP opening. Cells were treated with compounds at 0.001 μM for 15 min, and pore opening was induced with 1 mM TBHP. Calcein-AM and CoCl_2_ were used to measure mPTP aperture by flow cytometry. Cyclosporine A (CsA) at 0.2 μM was used as a positive control. Values are presented as the percentage of control cells. Data are mean ± SEM of three independent experiments. Statistical differences determined by one-way ANOVA and Dunnett’s tests. ^###^*p* < 0.001 compared to control cells. *p < 0.05 and **p < 0.01 compared to cells treated only with TBHP. (b) CypD expression after treatment with *S. tubulifera* metabolites. (c) Effect of compounds on the CypD expression under oxidative stress conditions. SH-SY5Y cells were treated for 6 h with **1**–**5** with and without 150 μM H_2_O_2_, and the expression of the protein was determined by Western blot. CsA at 0.2 μM was used as a positive control. Protein band expression was normalized by actin levels. Results are mean ± SEM of three replicates carried out in duplicate. Data expressed as the percentage of untreated control cells and H_2_O_2_ control. Values compared by one-way ANOVA and Dunnett’s tests. ^#^*p* < 0.05 and ^##^*p* < 0.01 compared to control cells. *p < 0.05 and **p < 0.01 compared to cells treated with H_2_O_2_ alone.

In view of the results obtained in the oxidative stress model and, in particular, in those related to mitochondrial functioning, the ability of compounds to inhibit mPTP opening was determined. For this assay, the minimal effective concentration in our previous assays was chosen (0.001 μM). SH-SY5Y cells were treated for 15 min with **1**–**5**, and 1 mM *tert*-butyl hydroperoxide (TBHP) was used to open the pore. Cells were loaded with calcein-AM, which accumulates in cytosol and cell compartments and with CoCl_2_ that quenches cytosolic fluorescence but not the mitochondrial signal.^17^ Then, fluorescence was analyzed by flow cytometry (Figure 6a). The addition of TBHP induced a significant drop in fluorescence (46.1 ± 8.2%, *p* < 0.001) due to mPTP opening. Cells treated with compounds **4** and **5** at 0.001 μM presented an increase in the fluorescence of 78.9 ± 6.3% (*p* < 0.05) and 90.3 ± 5.3% (*p* < 0.01), respectively, confirming the capacity of these metabolites to inhibit mPTP aperture. This augmentation was even higher than the increase produced by the known inhibitor of the pore CsA at 0.2 μM (77.6 ± 8.6%, *p* < 0.05).

Although the definitive biomolecular composition of mPTP has not been elucidated, CypD has been established as the key regulator of its opening.^7^ In this context, the expression of this protein was analyzed after treatment with compounds. CypD cytosolic levels were evaluated after treatment with **1**–**5** alone for 6 h and after the coaddition of diterpenes and H_2_O_2_ to get a complete insight into diterpene mitochondria-related effects. SH-SY5Y cells treated with **1**, **2**, **4**, and **5** at 0.001 μM presented a significant reduction in CypD expression, with levels among 64.8–71.9% of control cells, similar to the results obtained after the addition of 0.2 μM CsA (63.1 ± 10.0%, *p* < 0.05) (Figure 6b). Under oxidative damage conditions, the same diterpenes diminished CypD expression, being **2** the most effective one (37.9 ± 17.9%, *p* < 0.01). Compounds **1**, **4**, and **5** decreased the protein expression up to percentages of about 54% of H_2_O_2_ control cells (Figure 6c).

**Figure 7:**
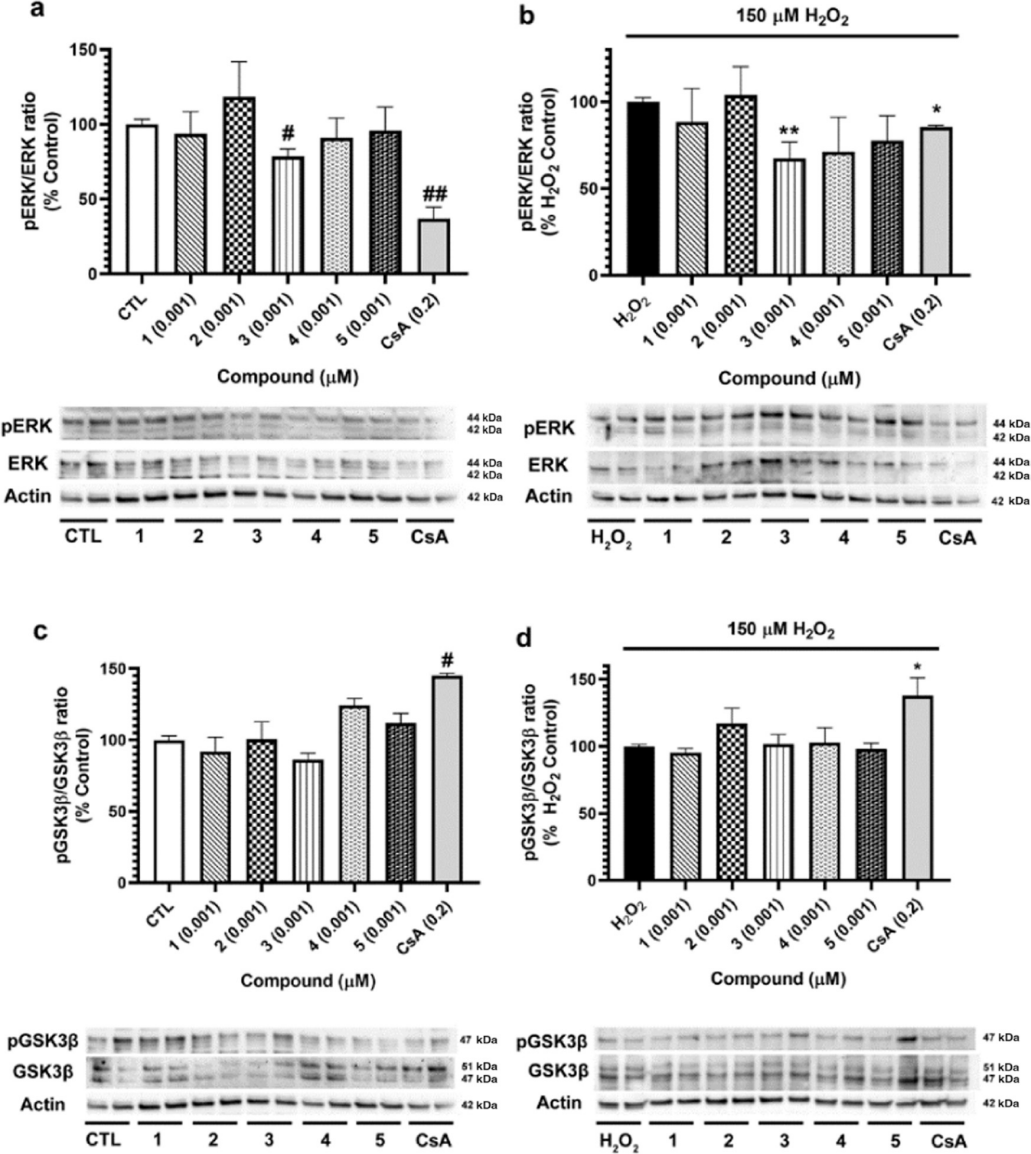
Analysis of compound effects on CypD upstream regulators. SH-SY5Y cells were treated with **1**–**5** at 0.001 μM for 6 h, and their effects on kinase activation were evaluated. (a) ERK activation after the addition of furanoditerpenes, (b) effect of compounds on ERK expression in oxidative stress conditions. (c) GSK3β expression after treatment with metabolites and (d) cotreatment with compounds and 150 μM H_2_O_2_. CsA at 0.2 μM was used as a positive control. Kinase expression was calculated as the ratio between phosphorylated and total levels. Protein band expression was normalized by actin levels. Results are mean ± SEM of three replicates carried out in duplicate. Data expressed as the percentage of untreated control cells and H_2_O_2_ control. Values compared by one-way ANOVA and Dunnett’s tests. ^#^*p* < 0.05 and ^##^*p* < 0.01 compared to control cells. *p < 0.05 and **p < 0.01 compared to cells treated with H_2_O_2_ alone.

Considering that CypD has several upstream regulators, which are also considered mPTP modulators,^10^ the study was continued by evaluating if *S. tubulifera* metabolites were affecting the activation of two kinases involved in the control of mPTP by CypD, ERK, and GSK3β (Figure 7). Regarding ERK, its activation has been related to the inhibition of CypD-mediated mPTP opening. Therefore, the phosphorylated state of residues implicated in ERK activation (Thr202, Tyr204, Thr185, and Tyr187) was determined, as well as its total levels. Any of the compounds were able to activate ERK under neither physiological nor oxidative conditions (Figure 7a,b). However, **3** inhibited the kinase in both situations, with levels of 78.6 ± 4.8% (*p* < 0.05) and 67.3 ± 9.4% (*p* < 0.01), respectively. CsA at 0.2 μM also reduced ERK phosphorylation in both cases, an effect previously described.^18,19^ On the other hand, GSK3β inhibition results in a reduction in mPTP opening since the kinase binds to CypD, decreasing its interaction with mPTP components.^10^ In the case of GSK3β, Ser9 phosphorylation was determined, a residue implicated in kinase inhibition (Figure 7c,d). No effects were found when neuronal cells were treated with compounds; only CsA displayed an increase in GSK3β phosphorylation.

**Figure 8:**
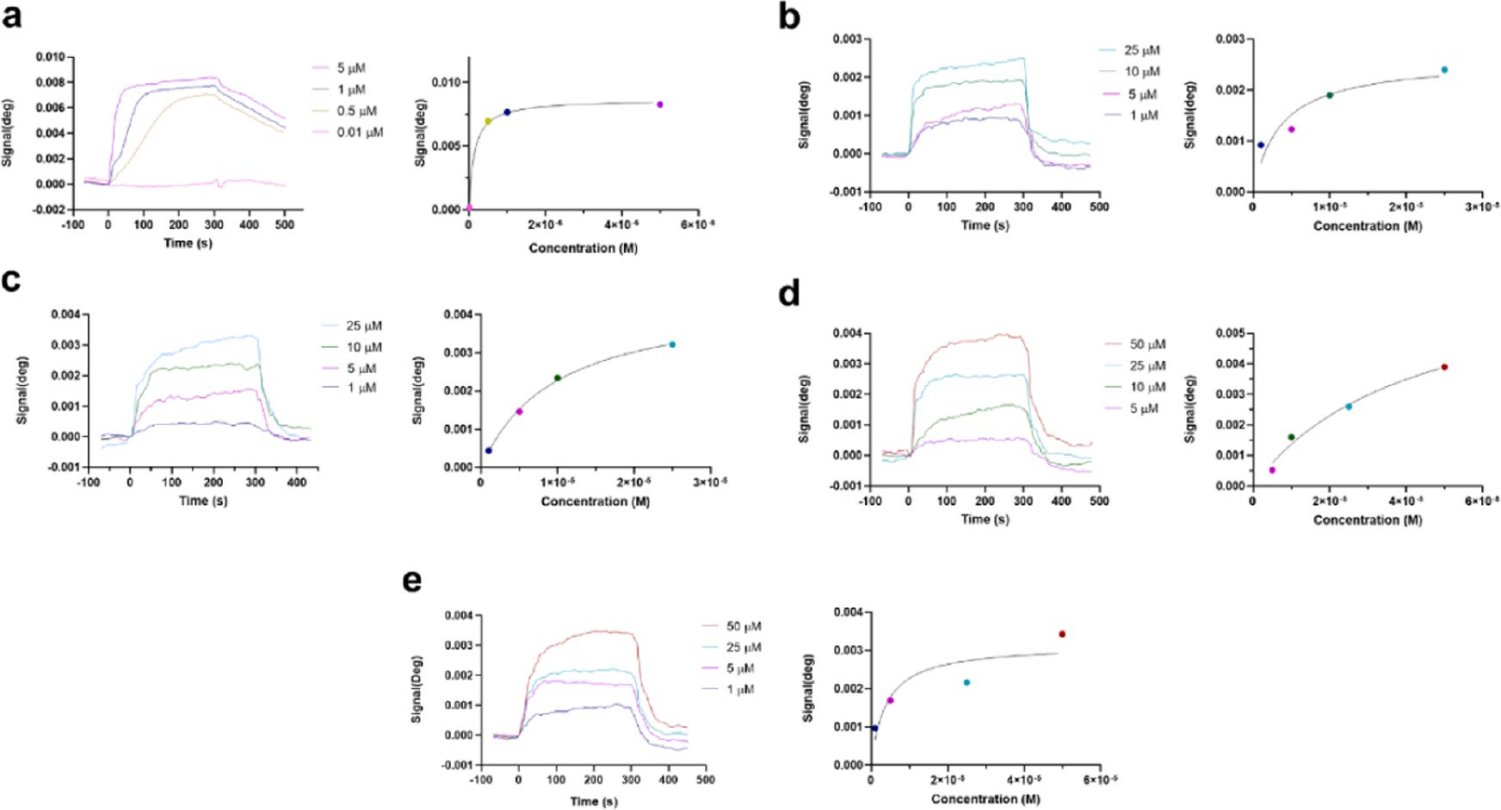
Binding of compounds to CypD measured by surface plasmon resonance. The left panels present association curves obtained by the addition of compounds over immobilized CypD and subtraction of their respective solvent control. The right panels show fitting curves for equilibrium binding. Representative graphs of one independent replicate. (a) Association of CsA, used as a positive control, and CypD, (b) compound **2**, (c) compound **3**, (d) compound **4**, and (e) compound **5** associations with the immobilized CypD. All injections were performed using PBS, 0.05% Tween (pH 7.2) as the running buffer. Kinetic equilibrium dissociation constants (*K*_D_) and fitting curves were obtained with TraceDrawer software.

Due to the lack of effects of diterpenes on CypD upstream regulators, we proceed to evaluate if compounds were capable of directly binding to the protein. With this objective, surface plasmon resonance (SPR) experiments were performed. CypD was immobilized in the sensor surface as a ligand, and **1**–**5** were injected as analytes. CsA was used as a positive control in the assays. For immobilization, amine groups were activated with 1-ethyl-3-(3-dimethylaminopropyl)carbodiimide hydrochloride (EDC) and *N*-hydroxysuccinimide (NHS) and 50 μg/mL of human CypD dissolved in 5 mM sodium acetate (pH 4.5) was added. The remaining active esters were inactivated with 1 M ethanolamine, and two injections of regeneration buffer (2.5 mM NaOH) were performed (Figure S6).^20^ No fall on the signal was observed, so compound injections were started. All of the assays were carried out with phosphate-buffered saline (PBS)—0.05% Tween (pH 7.2) as the running buffer, with a flow rate of 20 μL/min at 24 °C. At first, CsA at increasing concentrations was added to set the optimal conditions and to confirm the functionality of the protein. As can be observed in Figure 8a, dose–response association curves were obtained, with a maximum increase in the signal of 8.3 mdeg when 5 μM CsA was added. Individual curves were fitted with a 1:1 Langmuir model, obtaining a kinetic equilibrium dissociation constant (*K*_D_) of 0.13 ± 0.0003 μM. This constant is lower than previous values reported by our group due to the higher sensitivity of this instrument. In fact, in our previous assays, 5 μM was the lowest concentration used and no binding was detected, while it is the highest dose tested in these assays.^20,21^

Once the optimal conditions were established, diterpenes at different doses were added to CypD. No binding was detected with compound **1** up to 50 μM, whereas association curves for **2**–**5** were obtained (Figure 8b–e). Responses among 0.5 and 3.9 mdeg were observed when **2**–**5** were added at concentrations ranging from 1 to 50 μM. *K*_D_ was calculated for these metabolites, getting the following constants for compounds **2**–**5**: 2.53 ± 0.006, 7.00 ± 0.04, 30.4 ± 0.87, and 2.97 ± 0.01 μM, respectively. Therefore, diterpenes **2**–**5** are able to directly bind to CypD, so the capacity of compounds **4** and **5** to inhibit mPTP opening seems to be related to a direct binding to the protein. Kinetic parameters of CypD and compounds are summarized in Table S1.

### 2.3 Furanoditerpenes **1**–**5** Affect Cyps A–C

**Figure 9:**
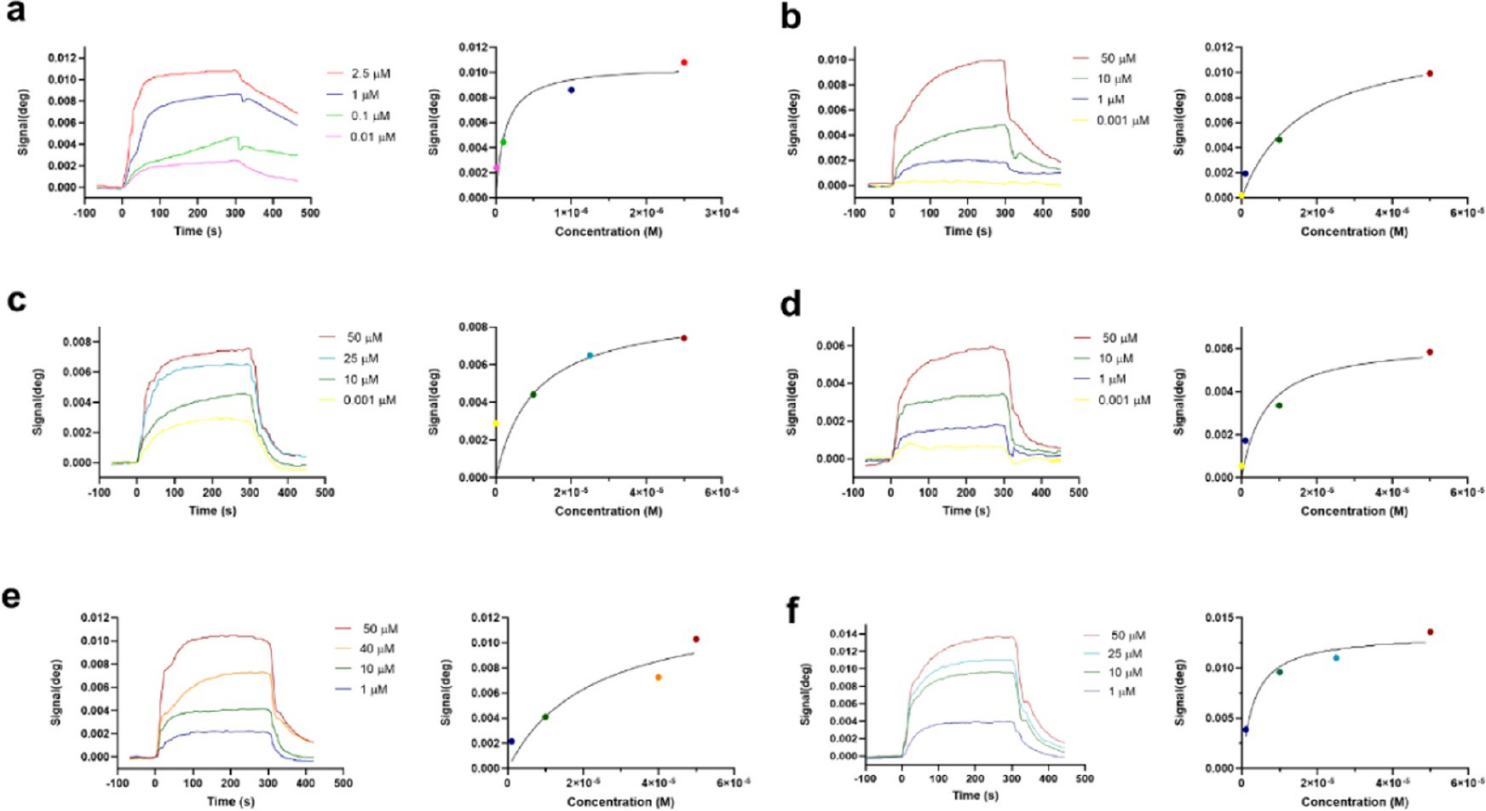
Binding of compounds to CypA measured by surface plasmon resonance. The left panels present association curves obtained by the addition of compounds over immobilized CypA and subtraction of their respective vehicle signal. The right panels show fitting curves for equilibrium binding. Representative graphs of one independent replicate. (a) Association between the positive control CsA and CypA. Association of (b) compound **1**, (c) compound **2**, (d) compound **3**, (e) compound **4**, and (f) compound **5** to immobilized CypA. Experiments were performed using PBS, 0.05% Tween (pH 7.2) as the running buffer. Kinetic equilibrium dissociation constants (*K*_D_) and fitting curves were obtained with TraceDrawer software.

Due to the affinity of furanoditerpenes by mitochondrial CypD, their ability to bind to CypA was determined to evaluate their selectivity between both isoforms. The protein was immobilized on the sensor surface by amino coupling, as described above (Figure S7), and compounds at several concentrations were added. The same conditions set for CypD were used for CypA experiments, with a flow rate of 20 μL/min and PBS-T (pH 7.2) as the running buffer. All assays were performed at 24 °C. Again, CsA was utilized as a positive control, being injected at concentrations between 2.5 and 0.01 μM. Typical association curves were obtained, reaching a response of 10.8 mdeg when the drug was added at 2.5 μM (Figure 9a). The *K*_D_ of CsA was calculated, and a value of 0.11 ± 0.0005 μM was obtained. This constant is almost equal to that calculated for CypD binding, confirming the validity of the experiments since CsA has a similar affinity for Cyps A and D^20,22^ and inhibits their isomerase activities in the same way.^23^ Therefore, assays with diterpenes were carried out. In this case, the five compounds were able to bind to CypA, obtaining dose-dependent association curves in all cases (Figure 9b–f). Diterpene **1** reached a signal of 9.9 mdeg at 50 μM, presenting a *K*_D_ of 15.2 ± 1.31 μM. Compound **2** presented a similar *K*_D_ (14.7 ± 1.08 μM), while **3** had a higher affinity to this isoform (*K*_D_ = 6.39 ± 0.09 μM). Finally, **4** showed responses between 0.2 and 10.3 mdeg, with a *K*_D_ of 27.4 ± 3.68 μM, and **5** presented a maximum signal of 13.6 mdeg (*K*_D_ = 4.34 ± 0.17 μM). Kinetic constants for CypA and compounds are presented in Table S2.

**Figure 10:**
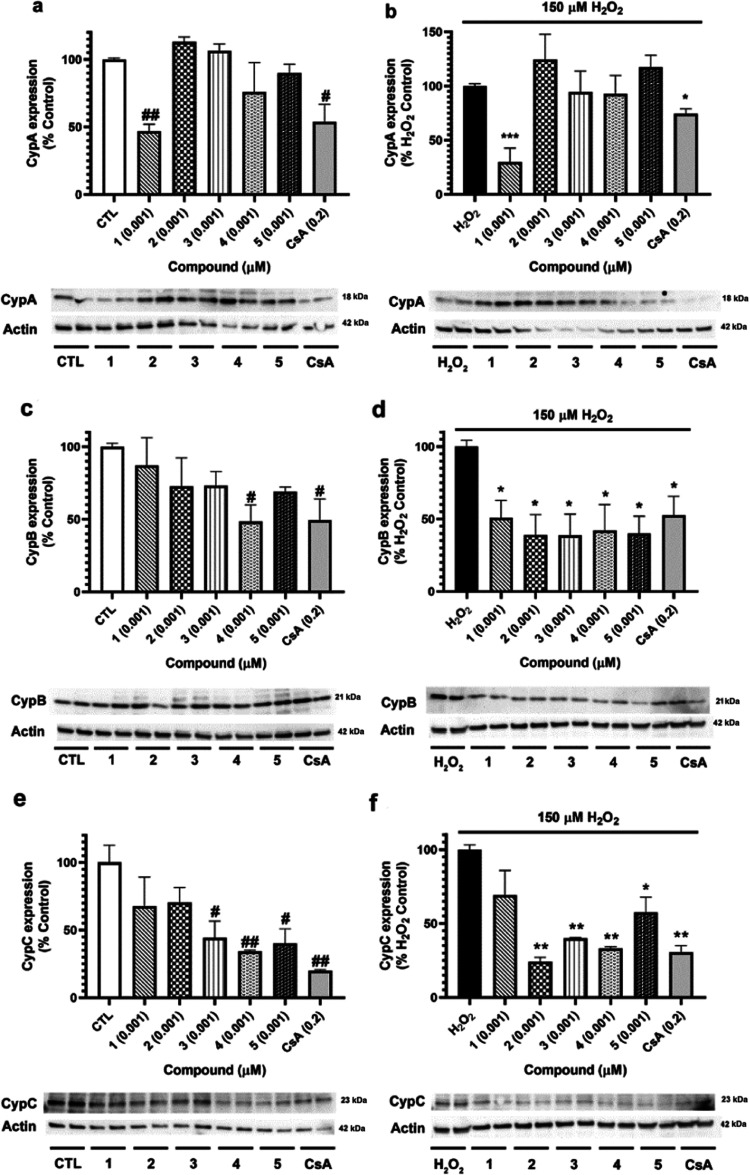
Cyps expression after the addition of furanoditerpenes. SH-SY5Y cells were treated with **1**–**5** at 0.001 μM for 6 h, and their effects on Cyps expression were determined by Western blot. (a) CypA cytosolic levels and (b) effect of compounds on CypA expression after oxidative damage. (c) CypB expression after treatment with metabolites, (d) CypB intracellular levels under oxidative stress conditions, (e) expression of CypC after the addition of *S. tubulifera* furanoditerpenes, and (f) CypC expression after cotreatment with metabolites and H_2_O_2_. CsA at 0.2 μM was used as the positive control. Protein band expression was normalized by actin levels. Results expressed as the percentage of untreated control cells or as the percentage of H_2_O_2_ control. Mean ± SEM of three replicates carried out in duplicate. Statistical differences determined by one-way ANOVA and Dunnett’s tests. ^#^*p* < 0.05 and ^##^*p* < 0.01 compared to control cells. *p < 0.05, **p < 0.01, and ***p < 0.01 compared to cells treated with H_2_O_2_ alone.

Since CypA seems to be a target of furanoditerpenes **1**–**5**, their effects on the expression of this protein, as well as in the intracellular levels CypB and CypC, the most studied isoforms of this immunophilin family, were evaluated. Before starting the experiments, antibody specificity was determined due to the conserved structures along Cyps. With this objective, 1 μg of human recombinant CypA, CypB, CypC, and CypD was subjected to electrophoresis and Western blot was performed as described for cell lysates (Figure S8). No cross-reaction was detected for anti-CypA, anti-CypB, and anti-CypC antibodies, but anti-CypD detected both CypA and CypD. Due to the differences in the molecular weight of these proteins (18 and 21 kDa, respectively), the discrimination between both bands was performed in cell lysates without uncertainty. Therefore, the assays were carried out. Human neuroblastoma cells were treated with compounds at 0.001 μM for 6 h, and Cyps expression was determined by Western blot (Figure 10). Regarding CypA, only compound **1** and CsA decreased its expression, both under physiological and oxidative stress conditions (Figure 10a,b). CypB expression was reduced by **4** until 48.54 ± 11.4% (*p* < 0.05) when cells were treated with metabolites alone, a comparable effect to that produced by CsA (50.2 ± 15.2%, *p* < 0.05) (Figure 10c). However, if oxidative damage was induced, all of the compounds generated a significant decline in CypB (Figure 10d), with levels of about 39–50% (*p* < 0.05) of H_2_O_2_ control. Finally, CypC intracellular levels were analyzed, finding a decrease in its expression after the addition of **3**–**5** alone (Figure 10e). The same compounds were able to reduce CypC levels when H_2_O_2_ was added to neuronal cells. Moreover, diterpene **2** also diminished the expression of this protein under oxidative injury, reaching a percentage of 24.3 ± 2.9% (*p* < 0.01) (Figure 10f).

### 2.4 Discussion

In this work, the mitochondrial-mediated neuroprotective effects of five furanoditerpenes obtained from *S. tubulifera* are described. Compounds presented promising activities against oxidative stress in human neuronal cells, mediated by their ability to bind CypD. Mitochondrial-targeted antioxidants have been proposed as a therapeutic strategy for the treatment of neurodegeneration, and some molecules such as MitoQ have even entered clinical trials.^24^ In this sense, furanoditerpenes **1**–**5** can be considered potential drugs for the treatment of neurodegenerative illnesses such as Alzheimer’s disease (AD), which currently does not have effective approved medicaments for counteracting its progression.^25^

Furanoditerpenes **2**–**5** displayed neuroprotective antioxidant properties against oxidative stress by decreasing the ROS release and recovering ΔΨ_m_ at nanomolar concentrations. Interestingly, compounds displayed a biphasic response, showing better outcomes at the lowest doses tested. This hormetic response has been described for several natural products, such as curcumin or resveratrol, which have neuroprotective properties at low concentrations and proapoptotic effects at high doses.^26,27^ This neuroprotective effect is related to the ability of *S. tubulifera* metabolites to bind CypD, as demonstrated by SPR assays. In fact, compound **1** was the less potent one in this *in vitro* model, being the only diterpene that did not show an affinity for CypD in biosensor experiments. Regarding CypD expression, a decrease was found in physiological conditions with compounds **1**, **2**, **4**, and **5**. This diminution in CypD expression after treatment with furanoditerpenes points to an enhancement of the protein degradation by compounds. The CypD complex with heat shock protein-90 prevents protein degradation, and their dissociation promotes CypD ubiquitination and proteasomal degradation.^28^ In this context, the binding of furanoditerpenes to CypD could be avoiding its association with the heat shock protein-90, triggering its degradation in the proteasome.

Although the best-characterized role of CypD in cells is the regulation of mPTP opening, its function goes beyond the pore, as has been recently discovered. CypD performs scaffolding functions, affects oxidative phosphorylation,^10^ and even modulates extra-mitochondrial signaling.^29^ In this context, the decrease produced by furanoditerpenes **1**, **2**, **4**, and **5** could be affecting mitochondria. However, the analysis of ROS levels and ΔΨ_m_ did not reveal any significant effect on the organelle function. The effects of furanoditerpene **1** on CypD intracellular levels point to an upstream target that could be associated with the protective effect produced by this compound since it did not present affinity to CypD. However, when ERK and GSK3β were evaluated, none of the compounds showed effects on these well-recognized CypD regulators. So, other enzymes that are known to modify the CypD activity such as sirtuin 3 or peroxisome proliferator-activated receptor α might be potential targets of **1**.^10^ On the other hand, compound **3**, with a *K*_D_ of 7.0 μM, did not affect CypD expression. This lack of effect could be related to the low dose used in the assay (0.001 μM); higher concentrations should be tested in future experiments.

The reduction produced in CypD expression by diterpenes **1**, **2**, **4**, and **5** under oxidative stress is very remarkable, as H_2_O_2_ increases the protein expression, making cells more susceptible to mPTP opening.^30,31^ To the best of our knowledge, the exact mechanism through which oxidative stress promotes the expression of CypD has not been described and transcriptional regulators of this protein remain elusive. Only a recent work has identified BMP/Smad signaling as a regulator of CypD gene expression,^32^ but its relationship to oxidative stress has not been explored.

CypD has been proposed as a target for neurodegenerative diseases, in which ROS increase and mitochondrial dysfunction are central pathological mechanisms.^16^ All compounds decreased ROS release, and **2**, **4**, and **5** were able to recover the GSH content. These effects are also related to their ability to target CypD, as the protein deletion attenuates H_2_O_2_-induced mitochondrial dysfunction and reduces intracellular ROS accumulation.^30^ Although mPTP allows the release of excessive ROS from mitochondria in normal conditions, under pathological circumstances, mPTP opening promotes ROS generation by mitochondria, generating a vicious cycle that is being interrupted by *S. tubulifera* metabolites. Surprisingly, only diterpenes **4** and **5** were able to inhibit mPTP opening after oxidative injury. This discrepancy can be associated with the differences in the incubation time in both experiments. CypD expression was evaluated after a cotreatment with compounds and the oxidant for 6 h, while cells were pretreated for 15 min with **1**–**5** followed by oxidative injury for 3 min for mPTP assessment.

CypD and mPTP involvement in neurodegeneration and more particularly in AD, the most prevalent neurodegenerative pathology, has been extensively studied. Elevated CypD expression has been detected in AD patients, and CypD-deficient mice presented improved memory.^33^ Moreover, a connection between tau and CypD has been recently established, observing that tau deletion leads to CypD downregulation and mPTP inhibition in a mouse model.^34^ With all of this evidence, the central role of CypD and mPTP in neurodegeneration is clear, so the discovery of compounds capable of targeting CypD and improving mitochondrial function is an important challenge. Despite the fact that CsA prevents mPTP opening, its use for the treatment of neurological pathologies has been discarded for many reasons, such as oxidative stress generation. CsA has immunosuppressant activity, it is not able to efficiently cross the blood–brain barrier, and it has shown neurotoxicity, nephrotoxicity, and hepatotoxicity.^35^

Interestingly, furanoditerpenes **1**–**5** showed different affinities for CypD and CypA. In all cases, binding among *S. tubulifera* metabolites and Cyps was reversible, as the injection of running buffer to the sensor was enough to dissociate the compounds from the proteins, which discards a covalent binding. Compounds **3** and **4** presented similar *K*_D_ values in both isoforms, whereas **1** was selective toward CypA and **2** and **5** displayed selectivity toward the mitochondrial isoform. These results suggest that the lack of a hydroxyl group at C-19 is critical for the selective affinity for CypA binding since it is the only difference between **1** and **4**. Moreover, the presence of a Δ^1^ double bond along with a hydroxyl group at C-2 and a ketone carbonyl functionality at C-3 seems to be responsible for the selective affinity toward CypD since all of these features are only present in **2** and **5**. In this sense, these furanoditerpenes could be useful as lead compounds to analyze the structural requirements for CypA or CypD affinity, as well as very helpful to better understand the implications of each Cyp on cellular signaling. In previous works, we have described a family of natural sponge diterpenes, the gracilins, with an affinity for CypA and CypD, which presented both neuroprotective and immunosuppressant properties.^20,22,36^ Interestingly, a pharmacophore-directed retrosynthesis led to obtaining simplified derivatives with selective mitochondrial-mediated neuroprotective activities.^21,37^ In this context, compounds **1**–**5**, bearing similar but more rigid structures to those of natural gracilins, could be used as scaffolds for new synthetic analogues, thus avoiding the supply problem of marine natural products.^38^ Moreover, rigid structures are very useful to obtain information about the shape of the target site and to determine the conformation adopted by a ligand when it binds to it.

Although furanoditerpenes **1**–**5** bind to CypA, only **1** reduced its cytosolic expression at 0.001 μM. This compound presented a *K*_D_ of 15.2 μM, showing lower affinity than **3** and **5** for CypA (*K*_D_ of 6.4 and 4.3 μM, respectively), but it was the only one with a selective affinity toward the cytosolic isoform. This selectivity could explain the effects of **1** at such a low concentration. Along with its function as a chaperone, the best-characterized role of CypA in cellular signaling is its interaction with calcineurin, which leads to the activation of calcineurin phosphatase activity and provokes interleukin-2 release. The inhibition of the CypA–calcineurin complex by CsA is responsible for the immunosuppressant properties of this drug.^39,40^ Despite the fact that **2**–**4** did not alter CypA intracellular levels in SH-SY5Y cells at 0.001 μM, these compounds bind to this isoform, so the effects of furanoditerpenes **1**–**5** on calcineurin pathway should be studied in the following works to clarify their potential immunosuppressant properties at higher doses. Anyway, their ability of binding CypA could also contribute to neuroprotection since this protein has also been linked to neurodegeneration. Although CypA is mainly located in the cytosol, it can also be released into the extracellular space, exerting proinflammatory activities. Extracellular CypA is a potent leukocyte chemoattractant and induces the expression of matrix metalloproteinases and cytokines.^41^ CypA has been involved in the blood–brain barrier dysfunction observed in AD patients, more particularly in apolipoprotein E4 carriers.^42,43^ Moreover, CypA colocalizes with Parkinson’s disease-associated protein α-synuclein,^44,45^ and targeting extracellular CypA has shown promising results against amyotrophic lateral sclerosis in a murine model.^46^

The function of the other immunophilins analyzed, CypB and CypC, has been less studied. CypB is located in the cytosol, the endoplasmic reticulum, and the nucleus, being also released to the extracellular space. Both pro- and anti-inflammatory activities of this isoform have been reported.^8^ Its overexpression in SH-SY5Y cells induced protective effects;^47^ however, CypB serum levels are elevated in metabolic syndrome patients and its intracellular levels are increased in liver and adipose tissue of obese mice.^48^ These tissues are of great importance at the beginning of obesity and type 2 diabetes, two known risk factors for dementia.^49,50^ With respect to CypC, it is located in the endoplasmic reticulum and the Golgi apparatus. This isoform participates in the redox regulation of endoplasmic reticulum, but its function remains elusive.^8,51^ It has been reported that CypC levels are augmented in microglial and neuronal cells after focal ischemia.^52^ Moreover, high serum CypC levels have been recently described in the acute coronary artery disease^53,54^ and its intra- and extracellular levels are elevated in activated human T lymphocytes.^55^ These recent results point to an important role of CypC in inflammation-mediated disorders such as neurodegenerative diseases.^56^

In conclusion, furanoditerpenes **2**–**5** isolated from *S. tubulifera* presented mitochondrial-mediated neuroprotective effects associated with their affinity to CypD. Specifically, compounds **4** and **5** were the most promising ones since they were capable of inhibiting mPTP opening at nanomolar concentrations. Furthermore, **1**–**5** presented different affinities for CypD and CypA related to their chemical structures, which turn them into potential scaffolds for the designing of new selective Cyps inhibitors useful for the treatment of disorders associated with inflammation and oxidative stress, such as neurodegenerative diseases.

## 3 Materials and Methods

### 3.1 Chemicals

TMRM, Thiol Tracker Violet, 5-(and-6)-carboxy-2′,7′-dichlorodihydrofluorescein diacetate (carboxy-H_2_DCFDA), MitoProbe Transition Pore Assay Kit, Pierce Protease Inhibitor Mini Tablets, Pierce Phosphatase Inhibitor Mini Tablets, Supersignal West Pico Luminiscent Substrate, and Supersignal West Femto Maximum Sensitivity Substrate were purchased from Thermo Fisher Scientific (Waltham, MA). Human CypD and CypA full-length proteins and CsA were from Abcam (Cambridge, U.K.). Recombinant human CypB was from Antibodies-online, and recombinant human CypC was obtained from BioVendor (Brno, Czech Republic). Compounds were dissolved in dimethyl sulfoxide (DMSO), and serial dilutions were performed in cell medium. Vehicle concentration was always kept under 0.5% in cell treatments, and control cells were treated with the highest concentration used to test its effects. Other chemicals were reagent grade and purchased from Sigma-Aldrich (Madrid, Spain).

### 3.2 Extraction and Isolation of Furanoditerpenes **1**–**5**

Compounds were obtained from the sponge *S. tubulifera* collected on the coast of the Mexican Caribbean. Samples were frozen, and extraction was performed with CH_3_OH–CH_2_Cl_2_. The organic extract was subsequently partitioned between H_2_O/CH_2_Cl_2_, and the CH_2_Cl_2_ portion was further fractionated into hexane, CH_2_Cl_2_, and aqueous methanolic fractions. The hexane fraction was submitted to silica gel flash column chromatography using a gradient mixture of hexane and EtOAc to afford enriched terpene fractions that were then submitted repeatedly to reversed-phase (RP) high-performance liquid chromatography (HPLC) separation (H_2_O/CH_3_OH mixtures) to yield **1**–**3**. Fractionation of the CH_2_Cl_2_ fraction by solid-phase extraction, using an RP-18 cartridge eluted with a stepped gradient from H_2_O, CH_3_OH, and CH_2_Cl_2_, yielded enriched terpene fractions. RP-HPLC separation of these fractions, using H_2_O/CH_3_OH mixtures, gave **4** and **5**. Full details on the isolation, purity, and structural elucidation of compounds have been published elsewhere.^2^

### 3.3 Cell Culture

Human neuroblastoma SH-SY5Y cells were purchased from American Type Culture Collection (ATCC), number CRL2266. Cells were cultured in Dulbecco’s modified Eagle’s medium: nutrient mix F-12 (DMEM/F-12) supplemented with 10% fetal bovine serum (FBS), 1% glutamax, 100 U/mL penicillin, and 100 μg/mL streptomycin. Cells were maintained at 37 °C in a humidified atmosphere of 5% CO_2_ and 95% air and dissociated weekly using 0.05% trypsin/ethylenediaminetetraacetic acid (EDTA). All of the reagents were provided by Thermo Fischer Scientific.

### 3.4 Cell Viability and Mitochondrial Membrane Potential Assays

To determine the neuroprotective effects of compounds, SH-SY5Y cells were seeded in 384-well plates at a density of 2.5 × 10^4^ cells per well. After 24 h, cells were treated with the metabolites at nontoxic concentrations (0.001–1 μM) and 150 μM H_2_O_2_ for 6 h. Vitamin E at 25 μM was used as a positive control. All of the experiments were performed as previously described.^15^

The ability of compounds to protect cell viability from H_2_O_2_ was evaluated with 3-(4,5-dimethylthiazol-2-yl)-2,5-diphenyltetrazolium bromide (MTT) test. After treatment, SH-SY5Y cells were washed three times with Locke’s buffer (154 mM NaCl, 5.6 mM KCl, 1.3 mM CaCl_2_, 1 mM MgCl_2_, 5.6 mM glucose and 10 mM *N*-(2-hydroxyethyl)piperazine-*N*′-ethanesulfonic acid (HEPES), pH 7.4). Then, cells were incubated with 500 μg/mL MTT for 1 h at 37 °C and 300 rpm. After this time, cells were solubilized with 5% sodium dodecyl sulfate. The absorbance of formazan crystals was measured at 595 nm with a plate reader. Saponin at 40 mg/mL was used as the death control, and its absorbance value was subtracted from the other data.

The effects of compounds in ΔΨ_m_ were analyzed with the fluorescent dye TMRM. For this assay, cells were washed twice with Locke’s solution and 1 μM TMRM was added to each well. After 30 min of incubation at 37 °C and 300 rpm, cells were lysed with H_2_O and DMSO at 50%. The fluorescence was read in a plate reader at 535 nm excitation and 590 nm emission. All of the assays were performed in triplicate three independent times.

### 3.5 Determination of Reactive Oxygen Species and Glutathione Intracellular Levels

For these experiments, cells were seeded in 384-well plates and treated as described above. All of the assays were carried out as previously described.^15^

The fluorescent dye 5-(and-6)-carboxy-2′,7′-dichlorodihydrofluorescein diacetate (carboxy-H_2_DCFDA) was used to evaluate ROS levels. After treatment with compounds and H_2_O_2_, cells were washed twice with serum-free medium. Next, cells were loaded with 20 μM carboxy-H_2_DCFDA dissolved in serum-free medium, and the plate was incubated for 1 h at 37 °C and 300 rpm. Then, 100 μL of PBS were added to each well for 30 min at 37 °C and 300 rpm. After this incubation, the fluorescence was read at 527 nm excitation, with an emission wavelength of 495 nm.

Thiol Tracker Violet was used to measure intracellular GSH levels, following the manufacturer’s instructions. Briefly, human neuroblastoma cells were rinsed twice with PBS and the fluorescence dye at 10 μM was added. The plate was incubated for 30 min at 37 °C and 300 rpm, and the fluorescence was measured at 404 nm excitation and 526 nm emission in a plate reader. All experiments were performed three independent times in triplicate.

### 3.6 Mitochondrial Permeability Transition Pore Assay

The inhibition of mPTP by compounds was determined with the MitoProbe Transition Pore Assay Kit, as previously described.^21^ For this assay, cells were seeded in 12-well plates at 5 × 10^5^ cells per well and allowed to grow for 24 h. Then, SH-SY5Y cells were detached with Detachin solution (Genlatis, San Diego), washed with PBS, and resuspended in PBS buffer with 0.6 mM CaCl_2_. Cells were loaded with 0.01 μM calcein-AM and incubated at 37 °C for 15 min. Then, 0.4 mM CoCl_2_ and compounds at 0.001 μM were added and cells were incubated for 15 min at 37 °C. After this incubation, cells were centrifuged, resuspended in 100 μL of calcium-free PBS, and kept on ice. Finally, TBHP at 1 mM was added for 3 min to the cells to induce the pore opening. Fluorescence was measured at 488 nm excitation and 517 nm emission wavelengths by flow cytometry using the ImageStream MKII instrument (Amnis Corporation, Luminex Corp, Austin). The fluorescence of 10 000 events was analyzed with IDEAS Application *vs* 6.0 (Amnis Corporation, Luminex Corp). Experiments were performed three independent times, and CsA at 0.2 μM was used as a positive control.

### 3.7 Western Blotting

SH-SY5Y cells were cultured in 12-well plates at 1 × 10^6^ cells per well and allowed to settle down for 24 h. Then, cells were treated with compounds at 0.001 μM for 6 h and oxidative damage was induced with 150 μM H_2_O_2_.

Cells were washed twice with ice-cold PBS, and a hypotonic solution was added (20 mM tris–HCl pH 7.4, 10 mM NaCl, and 3 mM MgCl_2_, containing phosphatase and protease inhibitor cocktail). Cells were incubated for 15 min on ice and centrifuged at 3000 rpm and 4 °C for 15 min. The supernatant was collected as the cytosolic fraction, and protein concentration was quantified with the Direct Detect instrument (Merck Millipore, Darmstadt, Germany).^15^

Electrophoresis was resolved in 4–20% sodium dodecyl sulfate polyacrylamide gels (Biorad, Hercules, CA), containing 15 μg of cytosolic protein from each sample. The Trans-Blot semidry transfer cell (Biorad) was used to transfer proteins to poly(vinylidene difluoride) (PVDF) membranes (Merck Millipore). Snap i.d. system (Merck Millipore) was used for membrane blocking and antibody incubation. CypD was detected with an anticyclophilin F primary antibody (1:1000, Abcam, cat. ab64935, lot. GR51090-7), phosphorylated ERK was quantified with antiphospho-ERK 1/2 (Thr202/Tyr204, Thr185/Tyr187) (1:1000, Merck Millipore, cat. #05-797R, lot. #2739182), and the total levels of the enzyme were detected with anti-ERK 1/2 antibody (1:1000, Thermo Fisher Scientific, cat. 13-600, lot. SE254471). Antiphospho-GSK3β (Ser9) (1:1000, Merck Millipore, cat. #05-643, lot. #2938844) was used to recognize phospho-GSK3β, and anti-GSK3β (1:1000, Merck Millipore, cat. #07-1413, lot. #3134282) was utilized to measure the total GSK3β expression. CypA was detected with anti-CypA primary antibody (1:1000, Elabscience, Madrid, Spain, cat. E-AB-15306, lot. DK0674), CypB was recognized with anti-CypB antibody (1:1000, Elabscience, cat. E-AB-22123, lot. AC0235), and CypC was quantified with anti-CypC antibody (1:1000, Proteintech, Manchester, U.K., cat. 10287-2-AP, lot. 00004874). Protein band intensity was corrected using anti-β-actin (1:10 000, Merck Millipore, cat. MAB1501, lot. 3698575). The activation of kinases was analyzed as the ratio between phosphorylated and total protein levels. Immunoreactive bands were detected with Supersignal West Pico Luminiscent Substrate and Supersignal West Femto Maximum Sensitivity Substrate. Diversity GeneSnap system and software (Syngene, Cambridge, U.K.) were used for protein band detection. Experiments were performed three independent times in duplicate.

### 3.8 Surface Plasmon Resonance Analysis

SPR measurements were performed using the MP-SPR Navi 210A VASA instrument with SPR Navi Control software (BioNavis, Tampere, Finland). All of the analyses were carried out in three-dimensional (3D) carboxymethyl dextran hydrogel-coated (CMD-3DM) sensors (BioNavis). PBS, 0.05% Tween (pH 7.2) was used as the running buffer for sensor activation, protein immobilization, and analyte injection. All buffers were degassed and filtered by 0.22 μm before use. The flow rate and temperature were set at 20 μL/min and 24 °C, respectively. A commercial Amine Coupling Kit (Xantec bioanalytics GmbH, Düsseldorf, Germany) was used for sensor activation and Cyps immobilization. The chip surface was preconditioned with 2 M NaCl and 0.01 M NaOH. Then, the surface was activated by the addition of 0.2 M 1-ethyl-3-(3-dimethylaminopropyl)carbodiimide hydrochloride (EDC) and 0.05 M *N*-hydroxysuccinimide (NHS). After activation, 50 μg/mL of CypA or CypD dissolved in 5 mM sodium acetate buffer (pH 4.5) were injected for immobilization on the sensor surface. The remaining reactive esters were deactivated by the addition of 1 M ethanolamine (pH 8.5).^20,22^ For binding measurements, analytes were added at concentrations between 0.001 and 50 μM for 5 min. Then, regeneration was performed by adding 2.5 mM NaOH for 1 min. CsA was used as a positive control in these experiments. Compound injections were performed in serial mode, and the reference channel signal was subtracted from the analyte sensorgram. Data were processed with MP-SPR Navi Data Viewer (BioNavis), and *K*_D_ values were obtained using the 1:1 Langmuir model with mass transport considerations of TraceDrawer software (RidgeView Instruments Ab, Uppsala, Sweden). All of the experiments were performed three independent times.

### 3.9 Statistical Analysis

Data are presented as mean ± SEM. Differences were evaluated by one-way ANOVA and Dunnett’s post hoc test with Graph Pad Prism 8.0 software. Statistical significance was considered at *p < 0.05, **p < 0.01, and ***p < 0.001.

## Supporting Information Available

^1^H NMR spectra of compounds, Cyps D and A immobilization over sensors, kinetic parameters of compound binding to Cyps and specificity of Cyps antibodies (PDF)
