# Supplementary material for: Furanoditerpenes from Spongia (Spongia) tubulifera Display Mitochondrial-Mediated Neuroprotective Effects by Targeting Cyclophilin D
Source: ACS Chem Neurosci. 2022 Jul 28;13(16):2449–63. doi: 10.1021/acschemneuro.2c00208 (PMC9686139; doi:10.1021/acschemneuro.2c00208)
Supplement: Supplementary file 1 — cn2c00208_si_001.pdf [file cn2c00208_si_001.pdf]

Supplementary Information

Furanoditerpenes from *Spongia (Spongia) tubulifera* Display Mitochondrial-Mediated Neuroprotective Effects by Targeting Cyclophilin D

Rebeca Alvariño[1, 2], Amparo Alfonso[1, 2], Dawrin Pech-Puch[3, 4], Sandra Gegunde[1, 2, 5], Jaime Rodríguez[3], Mercedes R. Vieytes[2, 6], Carlos Jiménez[3*], Luis M. Botana[1, 2*]

[1]Departamento de Farmacología, Facultad de Veterinaria, Universidad de Santiago de Compostela, 27002 Lugo, Spain

[2]Grupo Investigación Biodiscovery, IDIS, 27002 Lugo, Spain

[3]Centro de Investigacións Científicas Avanzadas (CICA) e Departamento de Química, Facultade de Ciencias, Universidade da Coruña, 15071 A Coruña, Spain

[4]Departamento de Biología Marina, Campus de Ciencias Biológicas y Agropecuarias, Facultad de Medicina Veterinaria y Zootecnia, Universidad Autónoma de Yucatán, 97100 Mérida, Yucatán, Mexico

[5]Fundación Instituto de Investigación Sanitario Santiago de Compostela (FIDIS), Hospital Universitario Lucus Augusti, 27002 Lugo, Spain

[6]Departamento de Fisiología, Facultad de Veterinaria, Universidad de Santiago de Compostela, 27002 Lugo, Spain

*Email: carlos.jimenez@udc.es, luis.botana@usc.es

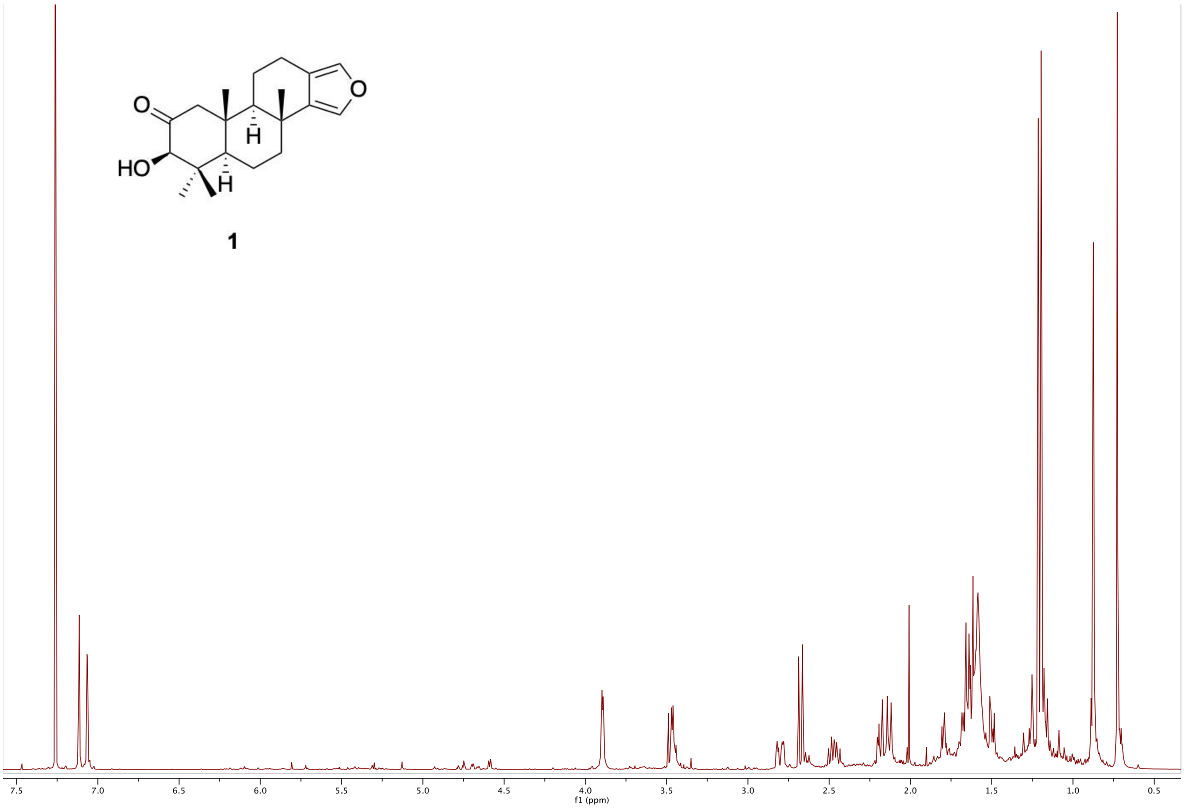

Figure S1. ^{1}H NMR spectrum of **1** (500 MHz, $CDCl_3$).

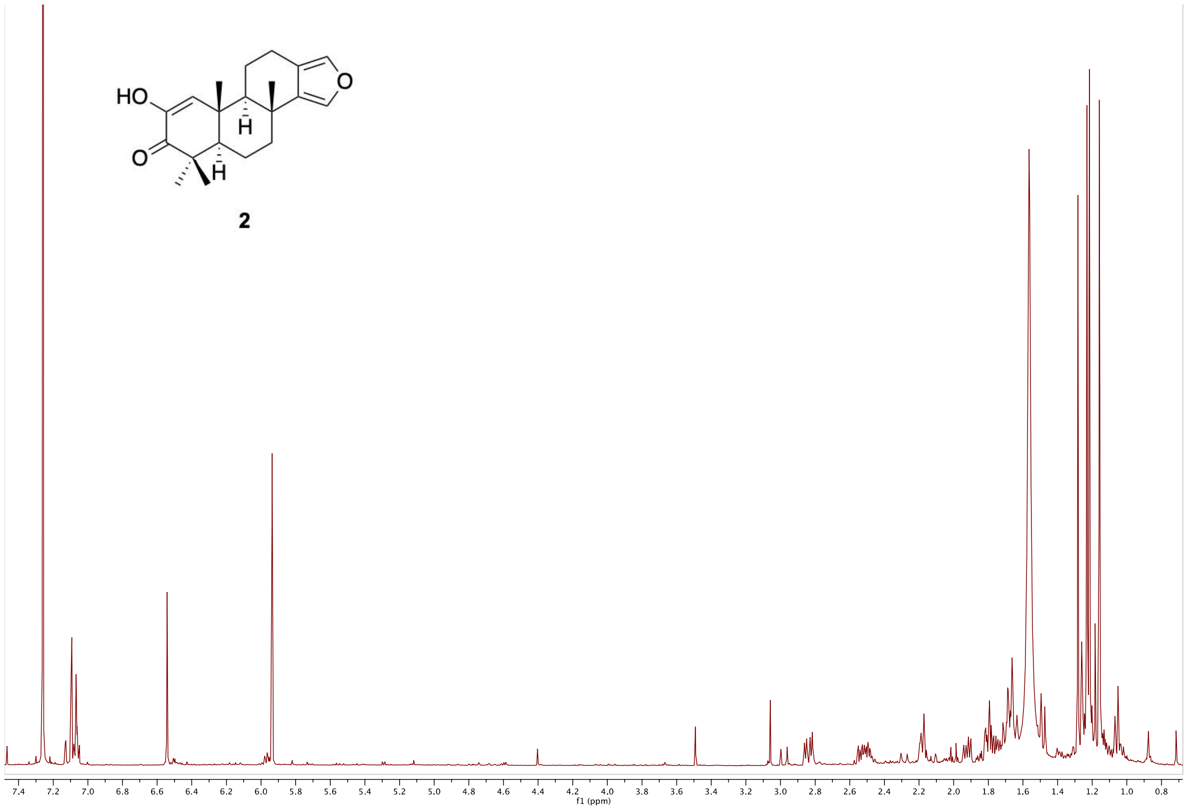

Figure S2. ^{1}H NMR spectrum of **2** (500 MHz, $CDCl_3$).

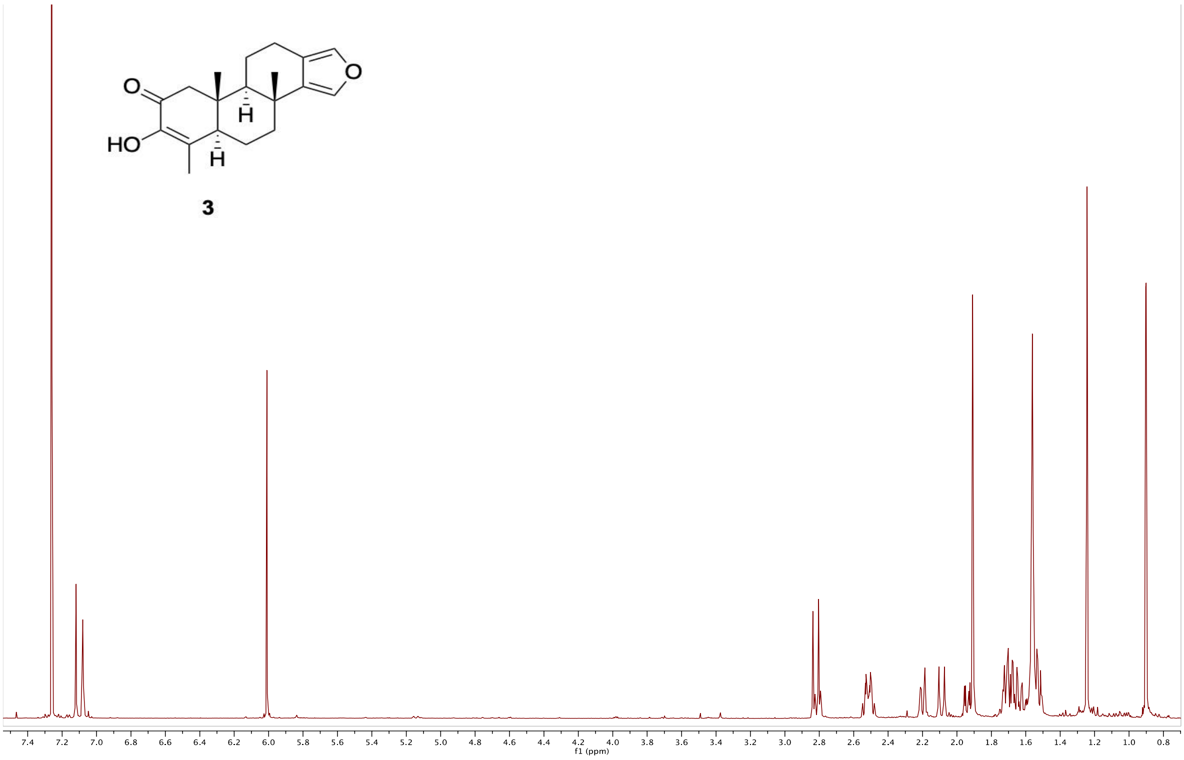

Figure S3. ^{1}H NMR spectrum of **3** (500 MHz, $CDCl_3$).

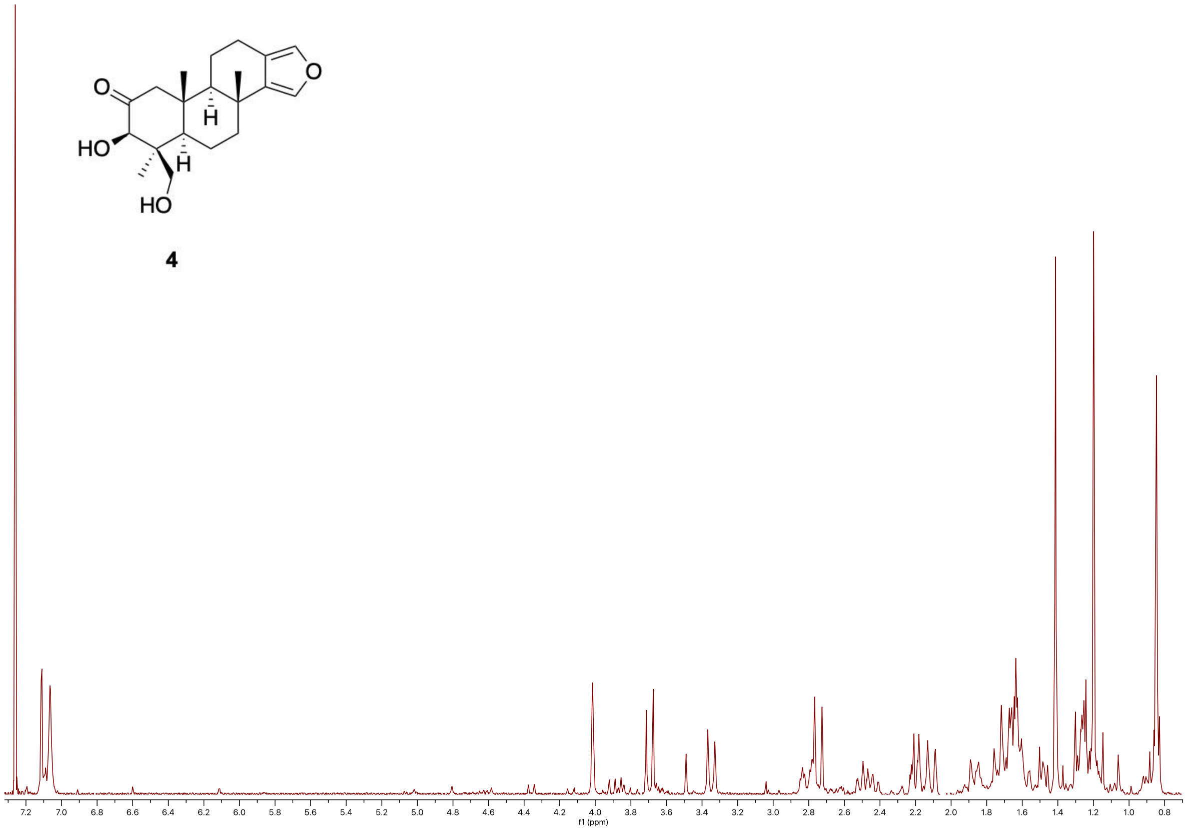

Figure S4. ^{1}H NMR spectrum of **4** (500 MHz, $CDCl_3$).

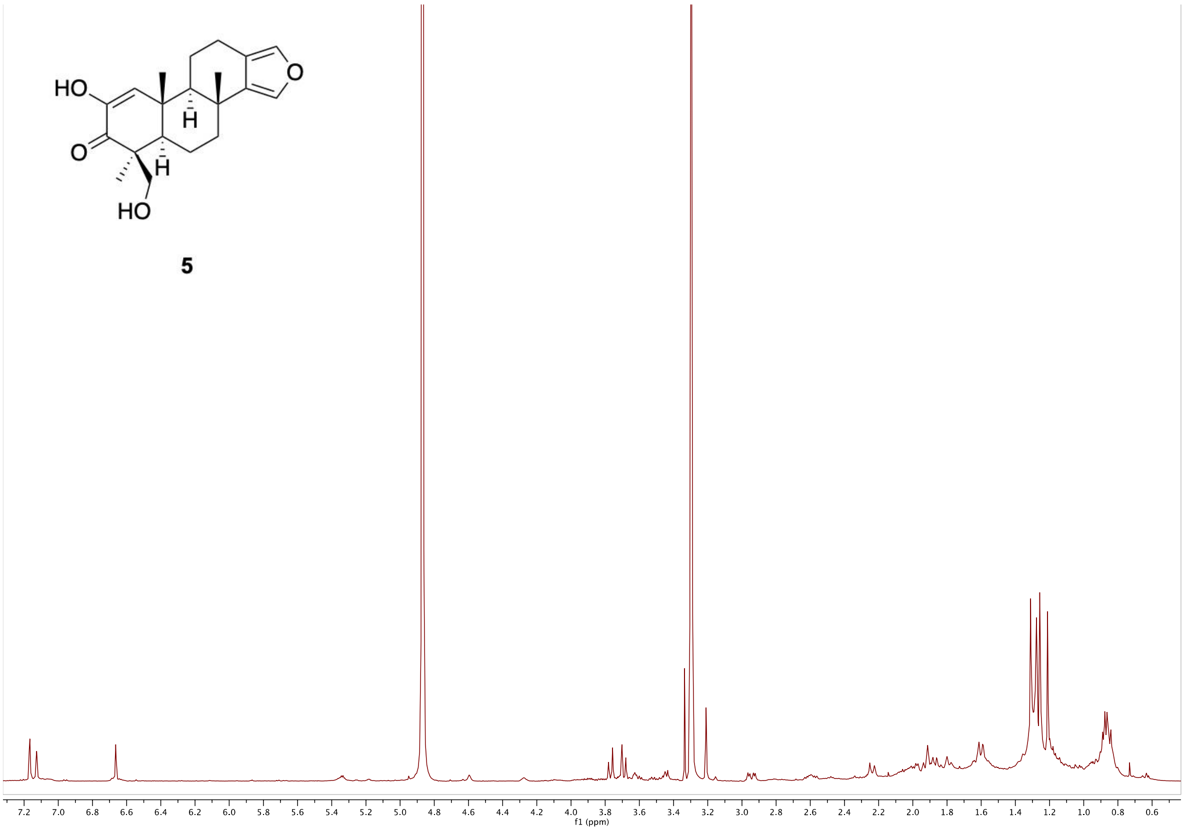

Figure S5. ^{1}H NMR spectrum of **5** (500 MHz, CD_3OD).

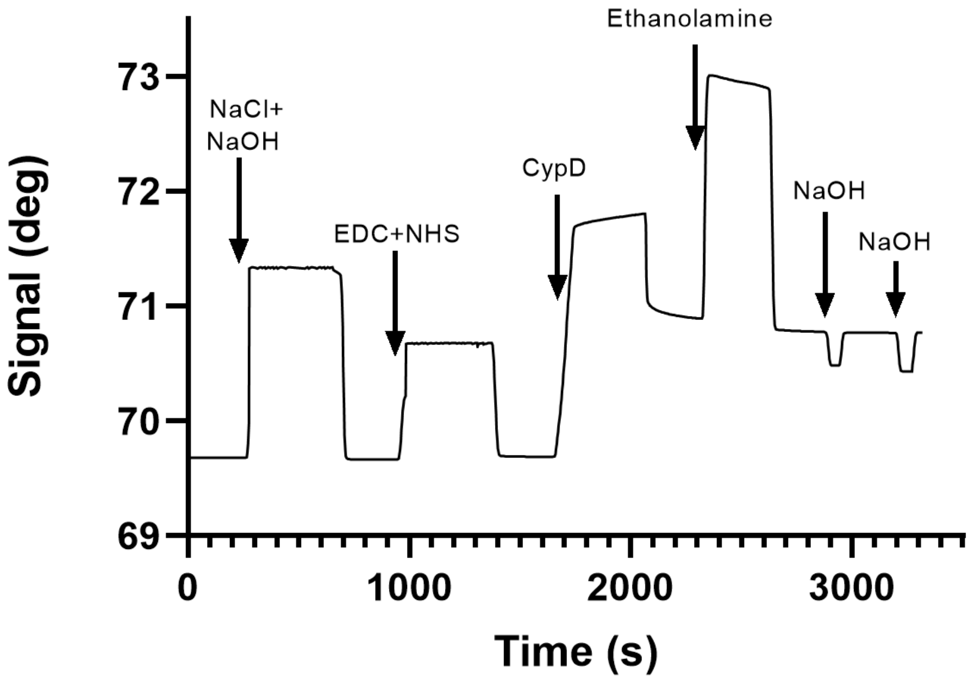

Figure S6. CypD immobilization over CMD-3D sensor. First arrow indicates pre-conditioning of surface with 2 M NaCl and 0.01 M NaOH for 7 min. Then, activation was done by addition of 0.2 M EDC and 0.05 M NHS for 7 min (second arrow). Third arrow shows injection of 0.05 mg/mL CypD dissolved in 5 mM sodium acetate (pH 4.5) for 7 min. Next, remaining activated groups were deactivated by injection of 1 M ethanolamine (pH 8.5) for 5 min, as indicated by fourth arrow. Finally, two regeneration injections were carried out (2.5 mM NaOH for 1 min) (fifth and sixth arrows).

Table S1. **Kinetic parameters of CypD and compounds binding.**

**Compound**	**Molecular weight (Da)**	***k*a (M s^{-1})**	***k*d (s^{-1})**	**K_D (μM)**
**CsA**	1202.63	1.59 10^4 ±3.8	2.08 10^{-3} ±3.8 10^{-8}	0.13±0.0003
**1**	316.20	No binding	No binding	No binding
**2**	314.19	1.00 10^4 ±2.3	2.53 10^{-2} ±2.3 10^{-8}	2.53±0.006
**3**	300.17	3.75 10^3 ±2.0	2.62 10^{-2} ±2.0 10^{-8}	7.00±0.04
**4**	332.2	7.38 10^2 ±2.1	2.24 10^{-2} ±2.0 10^{-8}	30.4±0.7
**5**	330.2	1.00 10^4 ±3.6	3.08 10^{-2} ±3.6 10^{-8}	2.97±0.01

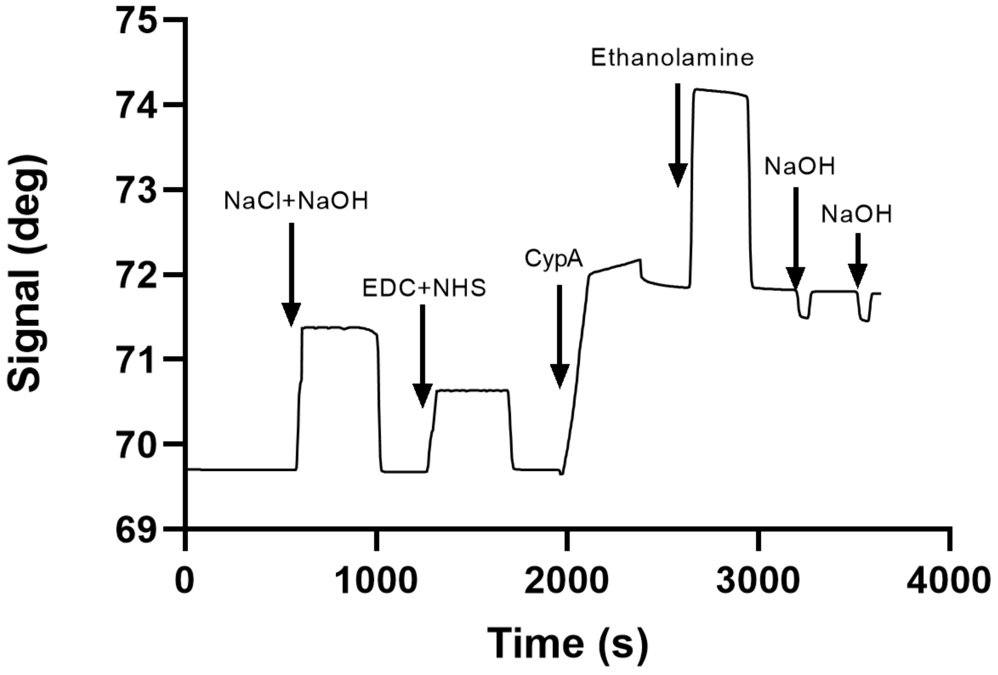

Figure S7. CMD-3D sensor activation and CypA immobilization. Firstly, sensor surface was pre-conditioned with 2 M NaCl and 0.01 M NaOH for 7 min (first arrow). Second arrow indicates the activation step (addition of 0.2 M EDC and 0.05 M NHS for 7 min).Then, CypA at 0.05 mg/mL dissolved in 5 mM sodium acetate (pH 4.5) was injected for 7 min. Fourth arrow indicates deactivation with 1 M ethanolamine (pH 8.5) for 5 min. Finally, 2.5 mM NaOH for 1 min was added twice (fifth and sixth arrows), without observing changes in the baseline.

Table S2. **Kinetic parameters of binding among CypA and compounds.**

**Compound**	**Molecular weight (Da)**	***k*a (M s^{-1})**	***k*d (s^{-1})**	**K_D CypA (μM)**
**CsA**	1202.63	2.16 10^4 ±1.1 10^2	2.33 10^{-3} ±1.1 10^{-7}	0.11±0.0005
**1**	316.20	6.82 10^2 ±5.8	1.04 10^{-2} ±5.6 10^{-8}	15.2±1.31
**2**	314.19	1.32 10^3 ±9.6	1.93 10^{-2} ±9.4 10^{-8}	14.7±1.08
**3**	300.17	3.19 10^3 ±4.3	2.04 10^{-2} ±4.3 10^{-8}	6.39±0.09
**4**	332.2	8.61 10^2 ±1.1	2.36 10^{-2} ±1.1 10^{-7}	27.4±3.68
**5**	330.2	3.59 10^3 ±1.4	1.56 10^{-2} ±1.4 10^{-7}	4.34±0.17

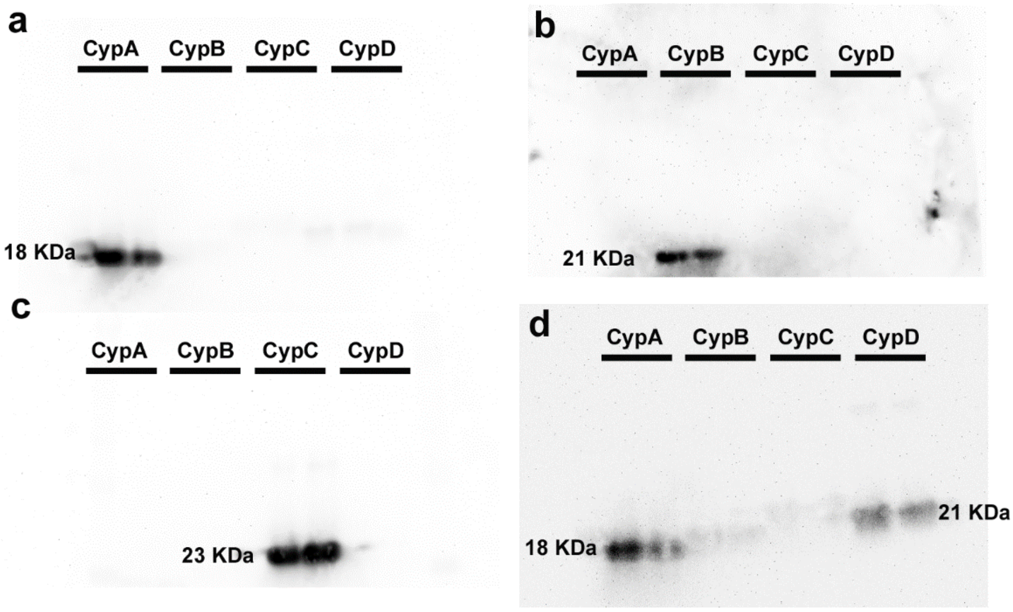

Figure S8. Specificity of Cyps antibodies. (a) Anti-CypA antibody, (b) anti-CypB antibody, (c) anti-CypC antibody and (d) anti-CypD antibody. 1 µg/mL of human recombinant CypA, CypB, CypC and CypD was loaded in a 4-20% SDS-PAGE gel, which was transferred to a PVDF membrane and specific antibodies for each Cyp were used. The same membrane was used for all the experiments, so stripping was carried out between the assays.
